# Bioimpedance Indices of Fluid Overload and Cardiorenal Outcomes in Heart Failure and Chronic Kidney Disease: a Systematic Review

**DOI:** 10.1016/j.cardfail.2022.08.005

**Published:** 2022-08-26

**Authors:** Kaitlin J. Mayne, Richard Shemilt, David F. Keane, Jennifer S. Lees, Patrick B. Mark, William G. Herrington

**Affiliations:** 1 Medical Research Council Population Health Research Unit, Clinical Trial Service Unit and Epidemiological Studies Unit, Nuffield Department of Population Health, University of Oxford, Oxford, UK; 2 Institute of Cardiovascular & Medical Sciences, BHF Glasgow Cardiovascular Research Centre, University of Glasgow, Glasgow, UK; 3 CÚRAM SFI Research Centre for Medical Devices, HRB-Clinical Research Facility Galway, National University of Ireland Galway, Galway, Ireland

**Keywords:** Bioimpedance, fluid overload, chronic kidney disease, heart failure

## Abstract

**Background:**

Bioimpedance-based estimates of fluid overload have been widely studied and systematically reviewed in populations of those undergoing dialysis, but data from populations with heart failure or nondialysis chronic kidney disease (CKD) have not.

**Methods and Results:**

We conducted a systematic review of studies using whole-body bioimpedance from populations with heart failure and nondialysis CKD that reported associations with mortality, cardiovascular outcomes and/or CKD progression. We searched MEDLINE, Embase databases and the Cochrane CENTRAL registry from inception to March 14, 2022. We identified 31 eligible studies: 20 heart failure and 11 CKD cohorts, with 2 studies including over 1000 participants. A wide range of various bioimpedance methods were used across the studies (heart failure: 8 parameters; CKD: 6). Studies generally reported positive associations, but between-study differences in bioimpedance methods, fluid overload exposure definitions and modeling approaches precluded meta-analysis. The largest identified study was in nondialysis CKD (Chronic Renal Insufficiency Cohort, 3751 participants), which reported adjusted hazard ratios (95% confidence intervals) for phase angle < 5.59 vs ≥ 6.4 of 2.02 (1.67–2.43) for all-cause mortality; 1.80 (1.46–2.23) for heart failure events; and 1.78 (1.56–2.04) for CKD progression.

**Conclusions:**

Bioimpedance indices of fluid overload are associated with risk of important cardiorenal outcomes in heart failure and CKD. Facilitation of more widespread use of bioimpedance requires consensus on the optimum device, standardized analytical methods and larger studies, including more detailed characterization of cardiac and renal phenotypes. (*J Cardiac Fail 2022;00:1 – 14*)

Heart failure and chronic kidney disease (CKD) commonly coexist but are often considered separately in research and clinical practice. The burden of heart failure increases with advancing CKD; the estimated prevalence of clinical heart failure is around 40% in patients requiring dialysis.^1–3^ Structural heart disease based on echocardiography is, perhaps, twice as common, with heart failure with preserved ejection fraction (HFpEF) the more frequent phenotype in CKD than heart failure with reduced ejection fraction (HFrEF).^2,3^ This interrelationship may be explained, in part, by shared risk factors but also by bidirectional etiological mechanisms. Heart failure increases the risk of CKD due to impaired perfusion of the kidneys and neurohormonal activation,^4,5^ and there are a number of pathophysiological changes associated with advancing CKD that contribute to heart failure. These include chronic hypertension and fluid overload as well as the possibility of direct uremia-related cardiotoxicity.^4^ Fluid overload is a common manifestation in both disease states and has clinical and prognostic implications. Treatment of both diseases has progressed with the advent of sodium-glucose cotrans-porter-2 inhibitors and mineralocorticoid receptor antagonists; both drug classes have diuretic mechanisms, and large trials have demonstrated benefits for cardiovascular risk and CKD progression.^6–9^

Fluid overload is traditionally assessed nonquantitatively by clinical examination.^10^ Bedside medical devices to standardize and more precisely quantify fluid status in both heart failure and CKD have been developed using ultrasound and bioimpedance technology. Both methods can be employed with relatively little training and allow rapid clinic-based measurements. Ultrasound modalities include lung ultrasonography^11–13^ and, less commonly, vascular ultrasound of the inferior vena cava and internal jugular veins.^12,14^ Bioimpedance methods include both bioimpedance analysis (BIA) (single-, multifrequency and bioimpedance vector analysis [BIVA]) and bioimpedance spectroscopy (BIS), both of which have been demonstrated to be highly reproducible and have been validated against gold-standard techniques^15^ more extensively than ultrasound approaches. Bioimpedance is a noninvasive measure of resistance and reactance of body tissues quantified by application of an electrical current via electrodes attached to the skin from which fluid compartment volumes and body composition can be estimated. Fig. 1 summarizes the 4 main bioimpedance approaches, including their analysis methods, commonly reported parameters and key advantages/disadvantages.

Although both BIA and BIS have been applied in heart failure and CKD, the Fresenius Medical Care (FMC) Body Composition Monitor (BCM), which uses BIS, is the most widely employed in patients with kidney disease because secondary calculations taking account of estimates of lean and adipose tissue mass (by applying a 3-compartment model)^16^ provide more specific estimates of fluid overload, independent of body composition (Fig. 1). Widespread measurement of BCM-determined fluid overload across the Nephrocare-FMC 26-country dialysis center network has facilitated large-scale observational studies, which have demonstrated strong positive associations with risk of all-cause mortality in patients requiring dialysis, independent of blood pressure.^17–19^ These studies used relative fluid overload derived by indexing absolute excess fluid volume to the volume of the extracellular water (ECW) compartment as the exposure, thereby allowing for comparisons between individuals.^20^ A threshold of > 15% relative fluid overload, equivalent to approximately+2.5 L absolute fluid overload,^20^ has often been used in analyses,^17–19^ whereas other studies have employed the more modest threshold of > 7% relative fluid overload (approximately equivalent to + 1.1 L absolute fluid overload).^19,21^ These dialysis studies have been subject to systematic reviews,^22–25^ but reviews of studies of heart failure and nondialysis CKD have not been reported. We sought to assess whether similar positive associations exist between fluid overload and adverse cardiorenal outcomes in other at-risk populations, where fluid overload may be less marked than in dialysis cohorts but may still represent a key modifiable cause of morbidity and mortality. A secondary aim was to identify a threshold of fluid overload, which is associated with adverse cardiorenal outcomes and which could be used as a surrogate marker of clinically significant fluid overload in both research studies and clinical care in heart failure and nondialysis CKD.

## Methods

The Preferred Reporting Items for Systematic Reviews and Meta-analysis (PRISMA) guidelines were followed, and the review was registered via PROSPERO international prospective register of systematic reviews (CRD42022316312). This report focuses on observational and interventional studies of adult populations with heart failure and/or CKD which have assessed the association between whole-body bioimpedance indices of fluid overload with risk of cardiorenal outcomes. Supplementary Table 1 summarizes the PI(E)COS (population, intervention [exposure], comparison, outcome, study design) framework applied in this review.

### Populations

Studies of patients with kidney failure requiring maintenance kidney replacement therapy were excluded. Studies exclusively of acute kidney injury and other acute disease states were also excluded (eg, sepsis, critical illness and perioperative studies), with the exception of acute decompensated heart failure. Studies of other chronic disease in which fluid overload may manifest (eg, liver disease) were also excluded.

### Exposures and Comparisons

All whole-body bioimpedance indices of fluid overload were considered relevant, including absolute and relative fluid overload (or overhydration), ratios of body water compartments, phase angle, vector length, and bioimpedance vector analysis (BIVA) hydration index, whether reported as continuous or categorical exposures. We tabulated results of both absolute fluid overload in liters and the related relative fluid overload parameter (indexed to measured ECW volume, expressed as a percentage)^20,26^ when both were reported.

### Outcomes

The primary outcome of interest was mortality (because the more specific outcome of cardiovascular mortality was not widely reported). Secondarily, we included studies reporting cardiovascular and kidney disease progression outcomes. For populations with heart failure, composite outcomes comprising all-cause death and hospitalization were included as a cardiovascular outcome on the presumption that a large proportion of deaths in these composite outcomes reflects cardiovascular disease in the included populations (and particularly in populations with heart failure).^4,27^

### Search Strategy

The systematic search was conducted within MEDLINE (Ovid), Embase (Ovid) and Cochrane Central Register of Controlled Trials (CENTRAL) from inception to March 14, 2022 (see Supplementary materials for search strategy). Search results were exported using Endnote software (EndNote X9, Clarivate, Philadelphia, US, 2013) and imported into Covidence software (Covidence, Veritas Health Innovation, Melbourne, Australia [no version number/date]) where duplicates were removed. Two reviewers (KJM, RS) independently screened all unique studies first by title/abstract, followed by a review of full texts for those studies that appeared potentially relevant; disagreement as resolved by consensus discussion.

### Data Extraction and Reporting

A bespoke Covidence electronic data extraction form was created for independent data extraction (KJM, RS); it included data fields for study design, funding, population characteristics, measures of kidney function and cardiac status, blood pressure and other laboratory parameters relevant to fluid overload at recruitment, as well as bioimpedance-outcome associations. Risk of bias was independently assessed by both reviewers using the Quality In Prognosis Studies (QUIPS) tool.^28^ To simplify presentation, for studies reporting multiple other fluidoverload exposures, our tabulations preferentially included the parameter most commonly reported across all studies unless, in our opinion, there were important differences in findings with less frequently used exposures. Results from multivariable confounder-adjusted models were emphasized wherever possible. Results from models that also included potential mediators of associations were extracted and are presented for comparison. Metaanalysis was considered but found not to be feasible (see Results).

### Thresholds for Clinically Significant Fluid Overload

Proposed thresholds for clinically significant fluid overload were developed through author expertise in bioimpedance plus review of the presented results in conjunction with reviews of data from dialysis studies^17–24,29–39)^ (see Discussion).

## Results

### Search Results

**Fig. 2:**
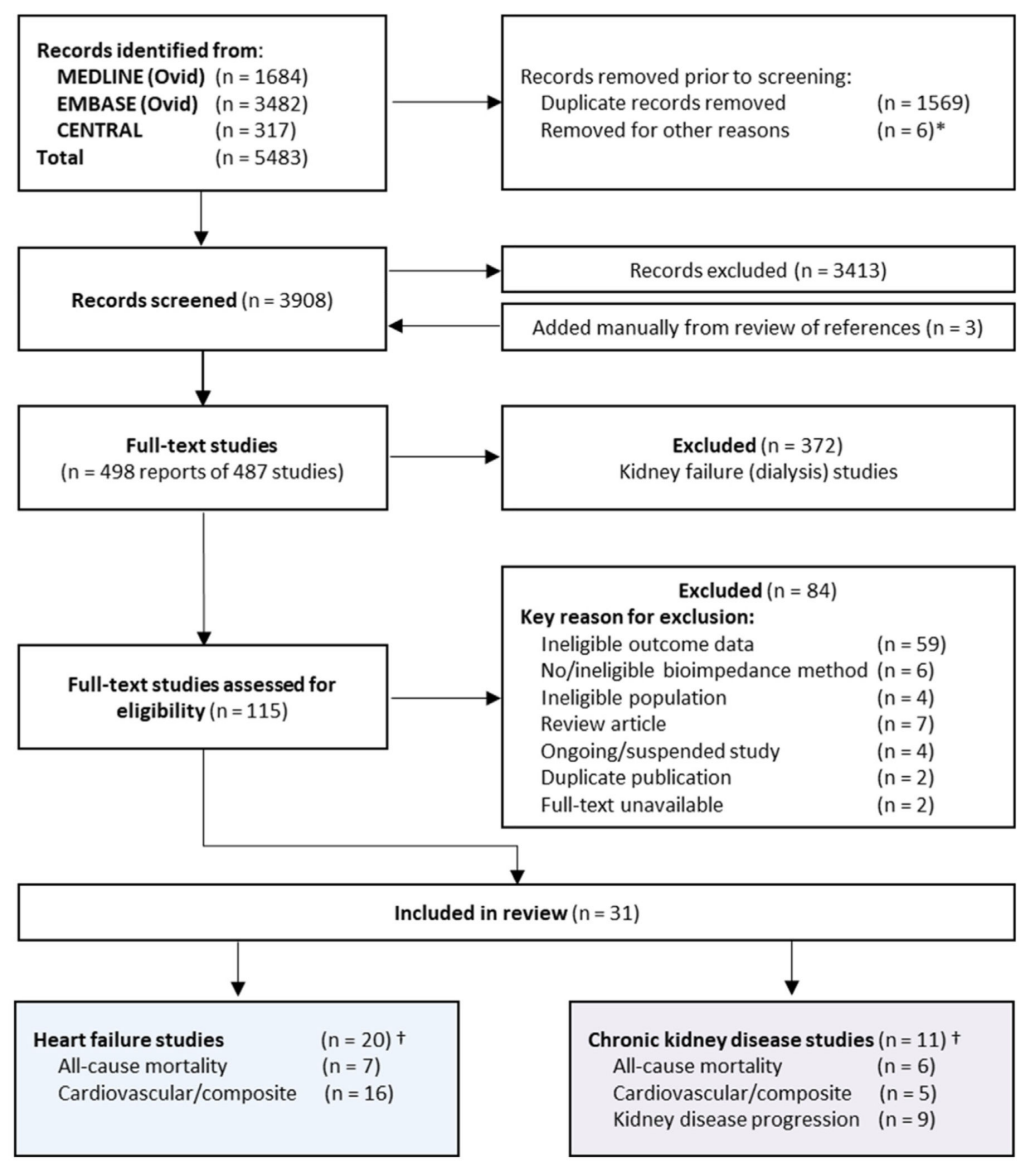
PRISMA flow diagram. *CENTRAL results produced 311 trials and 6 Cochrane reviews - 6 reviews removed as ineligible. † 6 CKD studies & 3 heart failure studies reported more than one relevant outcome.

Fig. 2 presents search results, reasons for exclusion, included studies, and reported outcomes. The final number of included studies was 31, of which 20 studied populations with heart failure,^40–59^ 10 studied populations with CKD,^60–69^ and 1 study included patients with type 2 diabetes with and without CKD.^70^ Methodological quality varied across studies; no studies were excluded due to high risk of bias. Risk of bias assessments are reported in Supplementary Table 7.

### Study Characteristics

Two studies included more than 1000 participants,^60,70^ but the majority of included studies were small. CKD studies were generally larger (range 100–3751 participants) than those in populations with heart failure (51–706 participants) and had longer durations of follow-up (range 1.0-8.6 years for CKD vs 0.02–3.0 years for heart failure cohorts). Heart failure studies more commonly studied participants with acute decompensated heart failure compared with stable chronic disease (Supplementary Table 4a) and heart failure subtypes (HFpEF vs HFrEF) were not frequently distinguished. Baseline characteristics were reported for the entire cohort in 71% (22/31) of studies (Supplementary Table 5a,b) and are summarized in Table 1. Average age ranged from 56–84 years (Supplementary Table 5a,b); the average proportion of male participants was 55% in both the heart failure and the CKD cohorts; and diabetes and hypertension were more common in the CKD cohorts than in the heart failure cohorts (diabetes: 45% vs 37%; and hypertension: 86% vs 78%, respectively). Ethnicity was not widely reported, although studies represent wide geographical coverage (Table 1). Confounding variables associated with CKD (such as albuminuria, CKD stage and measures of kidney function) were not widely reported in heart failure studies, and vice versa; baseline heart failure, left ventricular ejection fraction, New York Heart Association class and N-type brain natriuretic peptide (NTpro-BNP) were not widely reported in CKD studies (Supplementary Table 5a, b).

### Measurement of Fluid Overload

Fluid overload was assessed using 10 different bioimpedance parameters (8 parameters in heart failure and 6 in CKD); they are described in Supplementary Table 2. The most common parameters applied in CKD studies were absolute and relative fluid overload (also termed overhydration) as measured by the Fresenius BCM device. This device was used in only 2 (10%) heart failure cohorts (Supplementary Table 4a). BIA and BIVA devices were more commonly used in heart failure studies in which the BIVA hydration index was the most commonly reported parameter (Table 1).

The majority of studies (21 studies [68%]) reported single baseline measurements as opposed to serial measurements. Serial measurements were slightly more common in heart failure (7 studies [35%])^44,47,53,55,57–59^ than in CKD studies (3 studies [27%])^62,64,70^; serial measurements were commonly recorded over short timeframes during admissions due to heart failure. Reports tended to select preferentially a single exposure time point for observational analyses, relating fluid overload to future risk of outcomes, rather than considering time-updated exposures or applying adjustment for regression dilution bias.

### Mortality

Associations between fluid overload and specific causes of death were not widely reported in either heart failure or CKD cohorts, limiting the review to all-cause mortality. Associations between fluid overload with risk of death from any cause were presented in 13 studies, 10 of which reported estimates from multivariable models. Significant between-study differences in exposures and model approaches precluded meta-analysis.

Considering individual heart failure cohorts first: the largest studies demonstrated significant positive associations between bioimpedance indices of fluid overload and risk of all-cause mortality (Table 2). Massari et al. reported on 436 individuals, finding that a BIVA hydration index > 73.8% was associated with twice the risk of all-cause mortality compared to those with less fluid overload (adjusted hazard ratio [HR] 2.00, 95% confidence interval [CI] 1.20–3.20 [92 deaths]).^49^ Of note, Massari et al. included heart failure status (acute vs chronic), brain natriuretic peptide (BNP) and estimated plasma volume status (derived from hemoglobin and hematocrit, surrogate measures of intravascular volume status) in the multivariable model alongside the BIVA hydration index,^49^ meaning that models were estimating the relevance of total body fluid overload for a given level of intravascular status. The associations, therefore, estimate the relevance of excess extravascular fluid, rather than total body fluid overload, with risk. Similarly sized cohorts of populations with stable chronic heart failure^41^ and acute heart failure^51^ were studied for markers of total body fluid overload, and strong positive associations with risk of all-cause mortality were found, whether estimated by phase angle^41^ or BIVA hydration index^51^ (Table 2) (Supplementary Table 4a).

In CKD cohorts, the largest study, by Bansal et al. (3751 participants), demonstrated that phase angle < 5.59° (where lower phase angles represent higher degrees of fluid overload) vs ≥ 6.4° was associated with double the risk of all-cause mortality (HR 2.02, 95% CI 1.67–2.43 [776 deaths]) after adjustment for age, sex, ethnicity, and clinical site.^60^ Studies by Tsai et al. (236 participants in the included analysis) and Vega et al. (356 participants) using BCM-derived parameters were much smaller and, perhaps as a consequence, were unable to confirm statistically significant associations consistently (Tsai et al.: adjusted HR per % relative fluid overload 1.07, 95% CI 0.99–1.14 [23 deaths]; Vega et al.: adjusted HR per L absolute fluid overload 1.10, 95% CI 0.99–1.20 & HR per percentage of relative fluid overload 3.18, 95% CI 2.09-4.97 [113 deaths] (Table 2).^68,69^

### Cardiovascular Outcomes

Associations with composite cardiovascular outcomes were reported in 16 heart failure studies, 7 of which reported multivariable Cox regression analyses; a further 4 reported other multivariable regression analyses, and 5 reported only univariable associations (Table 3a). Five CKD studies reported relevant cardiovascular/composite outcomes (Table 3b). Composite cardiovascular outcomes in both heart failure cohorts and CKD cohorts commonly included death (all-cause, cardiovascular or cardiac) and hospitalization due to heart failure. CKD studies also often reported nonfatal myocardial infarction and stroke in cardiovascular composites (Table 3b). Substantial between-study differences in exposure definitions, modeling, ± outcome definitions again precluded statistical aggregation of study results.

Considering individual heart failure studies, 6 of the 7 studies that reported multivariable Cox models included hospitalization for heart failure in their composite cardiovascular outcome. Despite fewer than 100 of such outcomes in each study (Table 3a), all 6 reported statistically significant positive associations between increased baseline fluid overload assessed by a variety of parameters (BIVA hydration index in 3 studies^51,58,59^; ECW volume/ratio in 2 studies^47,53^; and relative fluid overload in 1 study^46^) and risk of these cardiovascular outcomes. The seventh study (by Lyons et al.^48^) reported on a composite of death, urgent transplant or ventricular assist device implantation and found no significant association between the ratio of ECW-to-total body water ≤ 0.39 vs ≤ 0.39 (adjusted HR 1.21, 95% CI 0.51–2.90; 56 outcomes). Adjustment for BNP and heart failure symptoms in this and other studies may result in models underestimating any causal relevance of associations, and for the majority of studies, we were unable to find less adjusted models, which are more relevant to the etiological scientific focus of this systematic review (Table 3a).

Of the 5 CKD studies reporting relevant cardiovascular/composite outcomes, the largest study reported a 48% (HR 1.48, 95% CI 1.15–1.91) increased risk of atherosclerotic cardiovascular disease (420 events, defined as incident myocardial infarction, ischemic stroke or peripheral arterial disease) and an 80% (HR 1.80, 95% CI 1.46–2.23) increased risk of heart failure events (581 events, not dependent on hospitalization; see Table 3b footnote for definition) for participants with phase angle < 5.59° (indicating higher level of fluid overload) vs ≥ 6.4° and after adjustment for age, sex, ethnicity, and clinical site.^60^ Notably, when additional variables such as albuminuria, blood pressure and serum albumin were added to the models—all factors that may mediate any causal effect between fluid overload and adverse outcomes—the associations were substantially attenuated, suggesting that these factors have key mediating contributions.^60^ Studies by Hung et al. and Tsai et al. also reported significantly increased risk of composite cardiovascular morbidity and mortality outcomes associated with fluid overload measured by the Fresenius BCM device, but were based on relatively small numbers of events (47 and 48 events, respectively)^63,68^ (Table 3b). Vega et al. reported only univariable analyses,^69^ and the final study by Ohashi et al. found a significant association between fluid overload and risk of all-cause hospitalizations (83 events) but not for the smaller number of cardiovascular outcomes (18 outcomes).

### Kidney Disease Progression

Progression to kidney replacement therapy initiation was reported in 4 studies, and a further 4 incorporated this into a composite outcome using percentage of estimated glomerular filtration rate (eGFR) decline (Supplementary Table 3). Two studies also included eGFR slope analyses.^63,68^ The largest studies consistently report increased risk of composite kidney outcomes associated with fluid overload as defined by absolute/relative fluid overload or phase angle^60,63,68,70^ (see Supplementary Table 3 for full details).

## Discussion

Whole-body bioimpedance is frequently used and well-studied in populations undergoing dialysis. In order to address the potential role of bioimpedance in heart failure and nondialysis CKD populations, we conducted a systematic review to summarize existing evidence and determine a threshold value of clinically significant fluid overload for use in research and clinical practice. We identified 31 eligible studies (20 heart failure and 11 CKD cohorts) which used 10 different fluid overload parameters derived from bioimpedance analysis or spectroscopy to assess associations with cardiorenal outcomes. Studies also varied greatly in size, duration, approaches to model construction, and outcome definitions, which precluded statistical aggregation of results by meta-analysis. Nevertheless, there was convincing evidence from individual studies that bioimpedance indices of fluid overload were associated with an increased risk of death in populations with both heart failure and CKD. Similarly, significant positive associations were observed with study-defined cardiovascular outcomes across the majority of studies. These associations appeared clearest for heart failure hospitalization outcomes, whereas evidence of a link with ischemic events were limited to CKD cohorts.

The findings from this systematic review are qualitatively consistent with the much larger body of evidence from dialysis populations.^22–24^ Such data are based largely on the Fresenius BCM device used in 7/11 [64%] CKD cohorts and 2/20 [10%] heart failure cohorts in our review. Dialysis studies have assessed a variety of threshold values of BCM-derived fluid overload. Wizemann et al. first established a 15% threshold value of relative fluid overload based upon the highest quartile of a reference hemodialysis population (measured predialysis),^20^ which was followed by studies of a > 7% threshold, derived from the 90th percentile of a healthy reference population.^21,33,71^ Both thresholds (or equivalents in liters) have been consistently linked to lower survival rates.^17–19,22,33,34,36–39,71^ Studies not using these thresholds selected cut-offs based upon quantiles of the study population, ranging between ≥ 4% and > 17.4%.^29–31,34,35^ In our review, we found no studies of heart failure or nondialysis CKD reporting associations with the 15% threshold value, perhaps because this degree of fluid overload is uncommon in earlier stages of CKD and heart failure compared with the extreme phenotype of fluid overload, which manifests in kidney failure requiring kidney replacement therapy. The 7% relative fluid overload threshold was applied in two CKD cohorts^63,68^ and 1 heart failure cohort^46^ and was positively associated with cardiorenal outcomes (Tables 2, 3a, 3b, and Supplementary Table S3). We, therefore, provisionally propose adoption of 2 levels of clinically significant fluid overload using BCM-derived measures: > 7% relative overload described as moderate and > 15% termed severe fluid overload. This is consistent with descriptors used by other authors^19,30,37,72^ and is the prespecified approach for analyses of an EMPA-KIDNEY trial substudy (ClinicalTrials.gov Identifier: NCT03594110)^73,74^ of ~ 650 participants with serial BCM measurements (see www.empakidney.org for data analysis plan).

A key advantage of the BCM over all other commercially available bioimpedance devices is its ability to quantify fluid overload independent of body composition (ie, lean and adipose tissue mass) by application of a 3-compartment model described by Chamney et al.^16^ It is not possible to equate BCM-derived fluid overload with other bioimpedance parameters, such as phase angle or BIVA hydration index, which were more commonly employed in heart failure cohorts (Table 1). Established BIVA hydration index reference ranges (fluid overload defined as hydration index > 74.3%^75^) were applied in the identified heart failure studies but, like phase angle and ECW ratios, this parameter may reflect differences in fluid volume, body composition or a combination of both. Multivariable analysis adjusted for body composition and nutritional factors may not completely address this limitation and is not practical for clinical application. For now, we propose that BCM measures are the optimum method to assess fluid overload for patients with heart failure and/or CKD.

Randomized evidence using whole-body bioimpedance indices to support clinical care have emerged from dialysis populations but there are limited randomized data from heart failure and nondialysis CKD populations. For example, in dialysis trials, bioimpedance-based assessment of fluid status vs standard clinical assessment improved parameters, such as blood pressure, left ventricular mass and arterial stiffness.^25,76–78^ This has yet to be shown to impact risk of hard clinical outcomes; randomized trials comparing bioimpedance added to standard care vs standard of care alone have not demonstrated meaningful impact on hospitalizations,^32,78^ preservation of residual kidney function,^79,80^ cardiovascular outcomes, or death,^32,77,78,81,82^ but numbers of outcomes in completed trials are generally small.^83^ Existing national clinical guidelines support the use of bioimpedance devices in dialysis patients when clinical assessment is challenging and suggest further consideration of the role of devices,^84,85^ though cost-effectiveness has not yet been demonstrated.^25^ Bioimpedance devices could be employed with a slightly different clinical aim in patients with earlier stage CKD not requiring dialysis. Fluid overload measured by bioimpedance is evident in very early CKD^86^ and has been associated with diastolic dysfunction^87^ and left ventricular hypertrophy on echocardiography.^88^ Identifying this subclinical diastolic dysfunction is challenging in CKD because NTpro-BNP is an imperfect diagnostic marker in those with decreased kidney function.^89^ Bioimpedance techniques may, therefore, represent an attractive tool for identification of patients with CKD who might benefit from screening echocardiographic assessments.

Bioimpedance technology has the potential to support clinical heart failure management by providing serial and objective assessments of fluid status with minimal between-operator differences, yet its use is not featured in recent international clinical guidelines.^90,91^ Bioimpedance devices have been shown to detect subclinical fluid overload^10,92^ which, in people with heart failure, is associated with increased risk of death or need for cardiac transplant.^93^ Bioimpedance may, therefore, support clinical decisions about when to intensify diuretic therapy so as to modify risk. Bioimpedance devices are generally portable and could be used in outpatient heart failure and CKD clinic assessments and even in patients’ homes. This strategy is being assessed in a small Korean pilot randomized trial assessing the impact of diuretic adjustment guided by home bioimpedance measurements vs standard care on change in NTpro-BNP and, secondarily, on risk of hospitalization for heart failure (NCT05177081). Segmental or localized impedance methods have also been tested and can be measured via implanted cardiac devices that quantify lung impedance. There is some evidence that fluid overload indicated by thoracic impedance predicts hospitalization and has the clinical potential to monitor diuresis.^94–96^ Nevertheless, we remain proponents of more widespread study and use of wholebody bioimpedance in a wider range of populations. There is a theoretical concern that whole-body bioimpedance devices may inhibit unipolar pacing in patients dependent on pacemakers, but the majority of pacemakers are now bipolar, and overall risk is considered low.

Our systematic review is the first to assess associations between bioimpedance indices of fluid overload and cardiorenal outcomes reported from heart failure and nondialysis CKD cohorts. The review has a number of limitations largely dictated by the nature of existing studies. First, the observational nature of the studies precludes causal inferences. Second, as described in Results, significant between-study differences in the fluid overload parameters and definitions of clinical outcomes precluded quantitative aggregation of results by meta-analysis. Furthermore, the wide range of reported models each considered a different set of covariates, often adjusting for combinations of potential confounders and mediators of associations simultaneously. This means models often addressed somewhat different research questions. Consequently, our review is limited to qualitative conclusions. Availability of individual participant data from included studies could address some of these limitations but would not address the differing approaches to fluid overload assessment or the relatively small size of completed studies. Third, studies commonly reported only single baseline bioimpedance measurements, which do not account for fluctuation in fluid status resulting in regression-dilution bias and reported associations underestimating the full importance of fluid overload in relation to outcomes. Last, studies rarely characterized both baseline and follow-up cardiac and CKD phenotypes, precluding the joint consideration of these overlapping populations.

In summary, whole-body bioimpedance indices of fluid overload appear to be consistently and positively associated with risk of death and adverse cardiovascular outcomes in populations with heart failure and nondialysis CKD, but there are limitations to the currently available evidence. Bioimpedance has several potential roles in clinical management and in clinical research in heart failure and nondialysis CKD. Its further development for these populations would benefit from consensus on the optimum device and standardization of analytical methods for such patients. Large studies recording serial measurements and more detailed baseline and follow-up characterization of both cardiac and renal phenotypes in a range of patients with heart failure and CKD are then needed to quantify more precisely and definitively any threshold above which fluid overload is associated with cardiorenal risks. Such studies could quantify the full extent and shape of associations and investigate the key potential mechanisms by which these associations are mediated.

## Supplementary Material

Supplementary material associated with this article can be found in the online version at doi:10.1016/j.cardfail.2022.08.005.

Supplementary material

## Figures and Tables

**Fig. 1:**
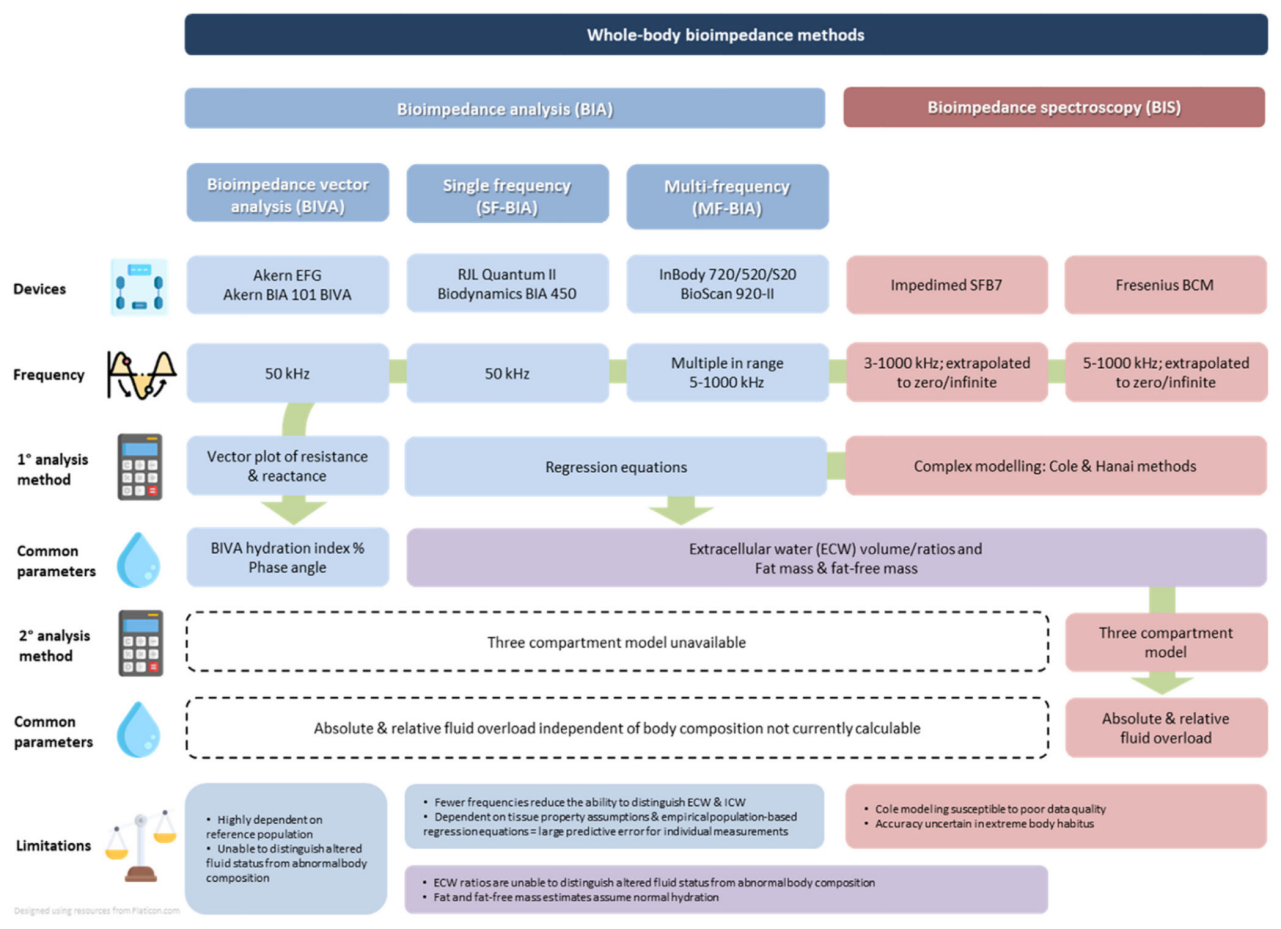
Whole-body bioimpedance methods. Only whole-body bioimpedance techniques are summarized, segmental approaches also exist. To the best of our knowledge, the BCM is the only commercially available device which applies the three compartment model. As indicated by the green arrow, vector plots, BIVA hydration index and phase angle can be derived by all devices; ECW ratios, fat and fat-free mass can only be derived from BIA & BIS devices.

**Table 1:** Summary of Cohort Characteristics

	Heart Failure Cohorts (n = 20)		CKD Cohorts (n = 11)	
Year of publication, n (%)				
2017–2021	11	(55)	8	(73)
2012–2016	8	(40)	2	(18)
Before 2012	1	(5)	1	(9)
Region, n (%)*				
Europe	12	(60)*	4	(36)
Asia	3	(15)	6	(55)
Russia	1	(5)	0	-
No. America	3	(15)*	1	(9)
So-. America	3	(15)*	0	-
Median number of total participants (IQR)	175(104–362)		236(177–347)	
Median follow-up (IQR), years	0.9(0.5–1.6)		3.3 (1.4–5.4)	
Median % male (IQR)	55 (49–63)		55 (50–61)	
Median % diabetes mellitus (IQR)	37 (35–44)		45 (36–49)	
Median % hypertension (IQR)	78(71–79)		86 (82–87)	
Bioimpedance method, n (%)				
Bioimpedance analysis (BIA)	7	(35)	4	(36)
Bioimpedance vector analysis (BIVA)	11	(55)	0	-
Bioimpedance spectroscopy (BIS)	2	(10)	7	(64)
Bioimpedance device, n (%)				
Fresenius Body Composition Monitor (BCM)	2	(10)	7	(64)
InBody S20/520/720	2	(10)	2	(18)
EFG	7	(35)	0	-
Quantum II/X	1	(5)	1	(9)
Other/not reported	8	(40)	1	(9)
Fluid overload parameter, n (%)				
Absolute & relative fluid overload	1	(5)	3	(27)
Absolute fluid overload	0	-	2	(18)
Relative fluid overload	1	(5)	1	(9)
Phase angle	3	(15)	2	(18)
BIVA hydration index/other BIVA	11	(55)	0	-
Extracellular water/ratio	4	(20)	3	(27)

Studies may report more than 1 fluid overload parameter; only those analyzed in association with clinical outcomes are presented (except for the related parameters absolute and relative fluid overload).

* Two studies included participants in 2 geographical regions.

**Table 2:** Associations Between Fluid Overload and Risk of All-Cause Mortality (Heart Failure and CKD Cohorts)

Author	Population	N	Follow-up (yrs)	Fluid overload definition	Baseline fluid overload Mean (SD)/ median (IQR)	n deaths	% deaths	Deaths/100 person yrs^1^	Analysis	Covariates						HR	95% CI LL	95% CI UL
										Age	Sex	DM	CVD	eGFR^2^	Other			
**Heart failure cohorts**																		
**Massari**, 2020	HF	436	1.3	BIVA hydration index (%) categories; >73.8%	73.7 (73.1-76.8) %	92	21	16.2	Cox MVSA	-	-	-	-	X	AHF vs CHF, BNP, estimated plasma volume status (based upon haemtocrit/haemoglobin)	2.00	1.20	3.20
**Colin-Ramirez**	HF	389	3.0	Phase angle (֯) Quartile 1 vs 4 (<4.2 vs ≥5.7)	5.0 (NA)֯^3^	66	17	5.7	Cox MVSA	X	-	X	-	-	Haemoglobin	3.08	1.06	8.99
**Nunez**	HF	369	1.0	BIVA hydration Index (%) >74.3% vs 72.7-74.3%	73.6 (73.0-76.2) %	80	22	21.7	Cox MVSA	Not reported In main publication						2.08	1.21	3.58
**Santarelli**, EHJ Acute CV Care	HF	336^4^	0.3	R/H, Xc/H Ω/m & BIVA hydration index (%); dR/H = change admission-discharge (median dR/H 11 Ω/m)	Xc/H: 36(14) Ω/m	33^4^	15	49.8	Cox MVSA^5^	“dR/H was associated with better prognosis (hydration Index 0.417, p<0.01)*^5^								
**De Berardinis**	HF	194	1.5	BIVA phase angle (°) Per - increment	4.4(1.7)°	47	24	16.2	Untvariable only: ROC. A∪C for death at 30 days: 0.64 (p=0.01) & 18 months: 0.86 (p<0.001), cut-off not given									
**Siriopol**	HF	151	1.7	Absolute fluid overload (L) Per 1L Increment	1.1 (2.8) L	53	35	20.7	Univariable only: Cox	-	-	-	-	-	-	1.11	1.02	1.19
				Relative fluid overload (%) Per % increment	4.8 (13.5) %	53	35	20.7	Univariable only: Cox	-	-	-	-	-	-	1.02	1.01	1.04
**Alves**	HF	71	2.0	Phase angle (°) <4.8 vs >4.8	5.6 (2.1) °	29	41	20.4	Cox MVSA	X	-	-	-	X	LV ejection fraction	2.67	1.21	5.89
**CKD cohorts**																		
**Bansal**	CKD	3751	7.0	Phase angle (°) Quartile 1 vs 3 & 4 combined (<5.59 vs ≥6.4)	6.6 (1.8)°	776	21	3.0	Cox MVSA	**X**	**X**				**Ethnicity, clinical site**	**2.02**	**1.67**	**2.43**
									Cox MVSA	X	X	X	X	X	uACR, BP, serum albumin, clinical site, ethnicity, smoking	1.31	1.09	1.58
**Vega**	CKD	356	4.2	Absolute fluid overload (L) Per 1L increment	0.6 (-0.4 to 1.5) L	113	32	7.6	Cox MVSA	X	-	X	X	-	Serum abumin, Charlson comorbidity, prealbumin, CRP	1.10	0.99	1.20
				Relative fluid overload (%) Per % increment	2.3 (0.8) %	113	32	7.6	Cox MVSA	X	-	X	X	-		3.18	2.09	4.97
**Tsai**	CKD	236	3.3	Relative fluid overload (%) Per % Increment	7.8 (8.6) %	23	10	3.0	Cox MVSA	X	X	X	X	X	uACR, medication (ACEI, ARB, diuretic, Statin), LDL	1.07	0.99	1.14
**Caravaca**	CKD	175	1.3	Phase angle (°) Per°increment	5.4(1.0)°	16	9	7.0	Cox MVSA	Not reported						0.49	0.26	0.92
**Ohashi**	CKD	149	4.9	ECW:ICW; assumed per increment (not specified)	NA	25	17	3.4	Cox MVSA	X	-	X	-	X	-	1.29	1.11	1.50
**Esmeray**	CKD	100	1.0	Absolute fluid overload (L) >0L vs ≤0L	NA	10	10	10.0	Univariable only: Kaplan-Meier. Cumulative survival significantly greater in ≤0L vs >0L, p=0.003 (not quantified)									

Lower phase angle indicates higher degrees of fluid overload. BIVA hydration index (%) ranges are based on standardized plots: hyperhydration >74.3%, normohydration 72.7%–74.3%, dehydration <72.7%. Where more than 1 multivariable model is presented with different levels of adjustment, the preferred model is highlighted in bold.

1 Event rate calculated for all studies from N, n and follow-up in years.

2 eGFR or other measure of kidney function.

3 50th percentile = 5.0°; IQR not reported.

4 33 deaths in 221 with AHF out of total 336 cohort (115 controls).

5 Cox MVSA results are not presented in tabular form; dR/H is the difference between R/H at admission and discharge; however, these results cannot be meaningfully interpreted.

ACEi, angiotensin-converting enzyme inhibitor; ARB, angiotensin-II receptor blocker; AHF, acute heart failure; AUC, area under the curve; BIVA, bioimpedance vector analysis; BNP, brain natriuretic peptide; BP, blood pressure; CHF, chronic heart failure; CKD,; chronic kidney disease; CI, confidence interval; CRP, C-reactive protein; CVD, cardiovascular disease; DM, diabetes mellitus; ECW, extracellular water; eGFR, estimated glomerular filtration rate; HF, heart failure; HR, hazard ratio; ICW, intracellular water; IQR, interquartile range; LL, lower limit; LV, left ventricular; MVSA, multivariable survival analysis; ROC, receiver operating characteristic; SD, standard deviation; uACR, urinary albumin:creatinine ratio; UL, upper limit.

**Table 3a:** Associations Between Fluid Overload and Risk of Cardiovascular Outcomes in Heart Failure Cohorts

Author	N	Follow up(yrs)	Fluid overload definition	Baseline fluid overload Mean (SD)/ median (IQR)	Outcome definition	n outcomes	% outcomes	outcomes\100 person yrs^1^	Outcome summary			Analysis	Covariates						HR/ OR	95% CI LL	95% CI UL
									Death	Heart	Other		Age	sex	DM	CVD	e GFR^2^	Other			
**Massarl, 2019**	706	0.02	BIVA HI (%), assumed per increment – not stated	77.7 (58) %	Length of stay Median 7.5 (7.4-8.1) days	NA	NA	NA	-	-	X	MV linear regression	-	-	-	-	X	BNP, NYHA, Hb, oedema	β 0.183	(p<0.001)	
**Di Somma, 2014**	381 ^3^	0.1	BIVA HI (%) >74.3% vs ≤74.3%	81.2(6.7)%	CV death^4^ or hospitalization	97	36	359. 3	X	-	X	MV logistic regression	X	-	-	-	X	BP	1.96	1.05	3.66
**Nunez**	369	1.0	BIVA HI (%) Per increment	73.6 (73.0-76.2) %	HHF	93	25	25.2	-	X	-	Cox MVSA; Fine & Gray^5^	Not reported						1.06	1.03	1.10
**Lyons**	359	2.1	ECW:TBW >0.39 vs 50.39^6^	NA	Death, urgent transplant, or VAD	56	16	7.4	X	-	X	Cox MVSA	X	X	X	-	X	BMI, HF etiology, NYHA BNP	1.21	0.51	2.90
**Santarelli**, EHJ Acute CV Care	336^7^	0.3	R/H, Xc/H Ω/m & BIVA HI (%); per increment	Xc/H: 36 (14) Ω/m	Death or hospitalization (presumed all-cause)	74	33	111-6	X	-	X	Unlvartable only Methods state Cox MVSA- not reported for death/rehospltallzatlon outcome; univariable analyses cannot be deaity Interpreted (ORs & ROC analysis reported stating Xc predicts events with an AUC 0.56, p=0.04 however cut-off value not gt✓en).									
**Santarelli**, Intern Emerg Med	292	0.3	BIVA HI (%), assumed per increment (not stated)	NA	CV death^4^	36	12	41.1	X	-	-	MV regression^8^	X	-	-	-	-	BNP, R,Xc, rales	1.10	0.97	1.25
**De Berardlnls**	194	1.5	Phase angle (°) Per °yyyy increment	4.4 (1.7)°	(1) Death or rehospitalization at 30 days^9^	40^9^	21	13.7	X	-	X	MV regression^9^	X	-	-	-	X	Beta blocker use, WCC, galectin-3	β-1.462	(p<0.03)	
			BIVA HI (%) Per increment	79.4 (6.6) %	(2) Death or rehospitalization at 30 days^9^	40^9^	21	13.7	X	-	X	MV regression’	X	-	-	-	X	Beta blocker use, WCC, gatectln-3	ß 0.103	(p≥0.05)	
**Sakaguchl**, 2015	190^10^°	0.5	ECW (L) measured/ predicted at discharge; per 0.1 unit	15.0 (5.5) L	Cardiac death^11^ or HHF	37	28	56.9	X	X	-	Cox MVSA	Not reported						1.48	1.20	1.83
**Liu**	159^12^	0.5	ECW:TBW predischarge^12^ per0.001 Increment	0.39(0.01)	HF events (death or hospitalization)	10^12^	9	18.9	X	X	-	Cox MVSA^12^	X	-	-	-	X	Allocation, Hb, uric acid, sodium, NYHA ACE1/ARB	1.06	1.02	1.10
**Koell**	150	2.0	Relative fluid overload (%); ≥7% vs <7%	NA	Cardiac death or HHF	51	34	17.0	X	X	-	Cox MVSA	X	X	X	-	-	BMI, 6-minute walking distance, NT-proBNP, AF	3.09	1.68	5.68
**Soloveva**	149	0.8	BIVA ‘congestion status at discharge per 1 rank’ (hydration index)	79.5 (6.5)%	(1) All-cause death or heart transplant	29	19	24.3	X	-	X	Untvariable only: Cox							1.73	1.23	2.45
					(2) All-cause death, heart transplant HHF	60	40	50.3	X	X	X	Untvariable only: Cox							1.40	1.10	1.79
**Trejo-Velasco**	105	0.9	BIVA HI (%); HI (<72.7 & >74.3%) VS 72.7- 74.3%^13^	NA	All-cause death or HHF	37	35	39.2	X	X	-	Cox MVSA	X	-	-	-	X	AF	2.60	1.05	6.44
**Sakaguchl**, 2020	100^14^	0.5	ECW (L), assumed per increment (not stated)	15.3 (6.9) L	Cardiac death^11^ or HF read mission	27	27	54.0	X	X	-	Untvariable only: Cox							0.96	0.89	1.04
**Curbelo**	99	1.0	Phase angle (°)	3.8 (1.5)°	HF events (death or hospitalization) Cardiac death or HF hospitalization	36	36	36.4	X	X	-	Untvariable only: ROC. AUC 57.0 95% Cl 43.2-70.8^15^									
**Villacorta**	80	0.6	BIVA HI (%) categories >74.3% at discharge	NA	Cardiac death^11^ or HF hospitalization	27	34	56.3	X	X	-	Cox MVSA	X	X	-	-	X	BNP, NGAL	1.39	1.25	1.54
**DI Somma**, 2010	51^16^	0.3	BIVA HI (%); cut-off >80.5%	79.0 (6.0) %	Death or re hospitalization for cardiogenic event	NA	NA	NA	X	-	X	Univariable only: ROC. Sensitivity 22%, specificity 94%, p=0.04 (no AUC)									

Lower phase angle indicates higher degrees of fluid overload. BIVA hydration index (%) ranges are based on standardized plots: hyperhydration >74.3%, normohydration 72.7%–74.3%, dehydration <72.7%.

1 Event rate calculated for all studies from N, n and follow-up in years.

2 eGFR or other measure of kidney function.

3 270/381 with AHF; 111 controls.

4 Not defined.

5 Unclear if HR from Cox or Fine and Gray analysis; reported in text only (supplement could not be obtained).

6 Manufacturer reference.

7 221/336 with AHF.

8 Unclear; ORs presented, Cox mentioned in methods-not reported.

9 10 deaths + 30 rehospitalizations at 30 days; MV regression analysis presented at 30 days only despite event numbers and ROC analysis at 18 months; death and rehospitalization are assumed to be all.

10 130 with AHF + 60 hospitalized controls; controls used to determine predicted values ECW only, analysis is of AHF patients (not compared to controls).

11 Death from HF, MI, sudden cardiac death.

12 53 in case management with BIA group; 53 in case management without BIA; 53 controls (routine care); MVSA is in 106 with EI measurements, event rate 10/106; BIA predischarge, 7 days postdischarge then monthly for 6 months.

13 Dehydrated and hyperhydrated groups combined in MVSA; HR not reported for hyperhydrated alone.

14 100 with central venous catheter and, therefore, included in survival analysis reporting fluid overload

15 Cut-off value not given.

16 25 AHF + 26 controls.

AF, atrial fibrillation; AUC, area under the curve; BMI, body mass index; CV, cardiovascular; Hb, hemoglobin; HHF, hospitalization for heart failure; HI, hydration index; MV, multivariable; NGAL, neutrophil gelatinase-associated lipocalin; NYHA, New York Heart Association class; R, resistance; TBW, total body water; VAD, ventricular assist device; WCC, white cell count; Xc, reactance.

**Table 3b:** Associations Between Fluid Overload and Risk of Cardiovascular Outcomes in CKD Cohorts

Author	N	Follow-up(yrs)	Fluid overload definition	Baseline fluid overload Mean (SD)/ median (IQR)	Outcome definition	Events Included In CV event/MACE definition													Covariates						HR	95% CI LL	95% CI UL
						n outcomes	% outcomes	Outcomes/100 person yrs^1^	Death	Nonfatal MI	Nonfatal	Angina	PCI	Heart failure	Arrhthymia	PVD	Other	Analysis	Age	Sex	DM	CVD	eGFR^2^	Other			
**Bansal**	3751	7.0	Phase angle (°); quartile 1 vs quartiles 3 & 4 combined (<5.59 vs ≥6.4)	6.6 (1.8)°	(1) Atherosclerotic CV disease	420	11	1.6	-	X	X	-	-	-	-	X	-	Cox MVSA	**X**	**X**				**Ethnicity, site**	**1.48**	**1.15**	**1.91**
																		Cox MVSA	X	X	X	X	X	uACR, BP, albumin, sito, ethnicity, smoking	1.12	0.86	1.45
					(2) Heart failure events^3^	581	15	2.2	-	-	-	-	-	X^3^	-	-	-	COX MVSA	**X**	**X**				**Ethnicity, site**	**1.80**	**1.46**	**2.23**
																		Cox MVSA	X	X	X	X	X	uACR, BP, albumin, site, ethnicity, smoking	1.03	0.82	1.29
**Vega**	356	4.2	Absolute (L)& relative fluid overload (%)^4^	0.6 (-0.4- 1.5) L 2.3 (0.8) %	CV events	150^5^	42	10.0	-	X	X	-	-	X^6^	-	X	-	Univariable logistic regreesion; not reported in table, text suggest no significant association with fluid overload and assume therefore not included in MVSA									
**Hung**	338	2.1	Absolute fluid overload (L); per 1L increment	NA	Composite CV morbidity & mortality	47	14	6.6	X	X	-	X	-	X^7^	-	-	-	Cox MVSA	**X**	**X**					**1.42**	**1.25**	**1.62**
																		Cox MVSA	X	X	X	X	X	BP, medication (ACEI, ARB)	1.28	1.09	1.50
																		Cox MVSA	X	X	X	X	X	uACR, BP, medication (ACEI/ ARB/dluretlc)	1.25	1.04	1.51
			Relative fluid overload (%); ≥7% vs ≥7%	8.3 (8.6) %	Composite CV morbidity & mortality	47	14	6.6	X	X	-	X	-	X^7^	-	-	-	COX MVSA	**X**	**X**					**6.22**	**2.78**	**13.92**
																		COX MVSA	X	X	X	X		BP, medication (ACEI, ARB)	3.84	1.68	8.76
																		Cox MVSA	X	X	X	X	X	uACR, BP, medication (ACEi, ARB)	2.71	1.14	6.48
**Tsai**	236	3.3	Relative fluid overload (%); per % Increment	7.8 (8.6) %	(1) CV events (MACE)	31	13	4.0	-	X	X	X	-	X^7^	X	-	-	Cox MVSA	X	X	X	X	X	uACR, medication (ACEi, ARB, statin diuretic), LDL	1.07	1.02	1.13
					(2) Composite MACE & all-cause mortality	48	20	6.2	X	X	X	X	-	X^7^	X	-	-	COX MVSA	X	X	X	X	X	uACR, medication (ACEi, ARB, statin diuretic), LDL	1.08	1,03	1.13
**Ohashi**	149	4.9	ECW:ICW; assumed per Increment (not specified)	NA	(1) CV events	18	12	2.5	-	X	X	-	X	X^7^	-	-	-	Cox MVSA	X	-	-	-	X	uACR, BP	1.12	0.93	1.31
					(2) Hospitalization (all-cause)	83	56	11.4	-	-	-	-	-	-	-	-	X	Cox MVSA	X	-	-	-	X	uACR, BP	1.18	1.08	1.28

Lower phase angle indicates higher degrees of fluid overload. Where more than 1 multivariable model is presented with different levels of adjustment, the preferred model is highlighted in bold.

1 Event rate calculated for all studies from N, n and follow-up in years.

2 eGFR or other measure of kidney function.

3 ”Heart failure events were determined based on clinical symptoms, radiographic evidence of pulmonary edema, physical examination of the heart and lungs, central venous hemodynamic monitoring data, and echocardiographic imaging in hospitalized patients based on the Framingham35 and ALLHAT36 criteria.”

4 Unclear which was used in CV event analysis, both are analyzed as continuous variables in all-cause mortality analysis.

5 150 participants experienced an event-total number of events not reported.

6 Heart failure defined as “presence of acute pulmonary oedema and an echocardiogram with ventricular systolic dysfunction and left ventricular ejection fraction <45” –does not specify hospitalization required.

7 Hospitalization for heart failure.

LDL, low density lipoprotein; MACE, major adverse cardiac events; MI, myocardial infarction; NOS, not otherwise specified; PCI, percutaneous coronary intervention; PVD, peripheral vascular disease.
