# Supplementary material for: Bioimpedance Indices of Fluid Overload and Cardiorenal Outcomes in Heart Failure and Chronic Kidney Disease: a Systematic Review
Source: J Card Fail. Author manuscript; Available in PMC 2022 Nov 8. (PMC7613800; doi:10.1016/j.cardfail.2022.08.005)
Supplement: Supplementary material [file EMS155997-supplement-Supplementary_material.pdf]

Bioimpedance indices of fluid overload and cardiorenal outcomes in heart failure and chronic kidney disease: a systematic review

Kaitlin J. Mayne, Richard Shemilt, David F. Keane, Jennifer S. Lees, Patrick B. Mark, William G. Herrington

Supplementary Materials

Contents

Supplementary Methods 2
Abbreviations used in Supplementary Tables 3
Supplementary Table S1: PI(E)COS framework 4
Supplementary Table S2: Summary of bioimpedance indices of fluid overload employed in included studies 5
Supplementary Table S3: Associations between fluid overload and risk of kidney disease progression 6
Supplementary Table S4a: Study characteristics for all included heart failure cohorts 7
Supplementary Table S4b: Study characteristics for all included CKD cohorts 8
Supplementary Table S5a: Baseline participant characteristics from heart failure cohorts 9
Supplementary Table S5b: Baseline participant characteristics from CKD cohorts 10
Supplementary Table S6: Studies with multiple reports identified 11
Supplementary Table S7: Risk of bias (ROB) assessment for all included studies 12
Quality in Prognostic Studies (QUIPS) tool for risk of bias 13
MEDLINE (Ovid) search strategy 15
Kidney disease outcome nomenclature 17
References 18

Supplementary Methods

Revised protocol

The original protocol included kidney failure populations however this decision was revised based upon the number of studies retrieved. Associations in dialysis (kidney failure) populations have been established by existing reviews therefore we focused this review on non-dialysis CKD and heart failure populations. The initial title/abstract screening was performed in two stages: firstly screening all studies and then secondly removing all retained studies which clearly studied only dialysis (kidney failure) populations. Records of these studies were retained for review of fluid overload definitions and threshold values in existing studies across all CKD (non-dialysis and dialysis) and heart failure populations.

Search strategy

Studies of potential relevance not identified by the systematic search (through hand searching of references of relevant studies) were added prior to screening. The database search was not restricted by language however all studies were available in English. Conference abstracts were excluded. Studies were only included once and the approach to selection of the report used for extraction was based upon the following factors: maximal outcome data, maximal follow-up time and largest population. Where different fluid overload parameters were used, this was considered alongside the aforementioned factors and parameters most synonymous with other studies were favored.

Eligibility

Fluid overload measurements at any time point and studies reporting both single and serial measurements were included. Studies using only segmental/localized (as opposed to whole-body) bioimpedance such as intra/transthoracic or calf measurements were excluded. Where eligibility was unclear, authors were contacted by email.

Abbreviations used in Supplementary Tables

ACEi	Angiotensin-converting enzyme inhibitor
AHF	Acute heart failure
ARB	Angiotensin-II receptor blocker
BCM	Body composition monitor
BIA	Bioimpedance analysis
BIS	Bioimpedance spectroscopy
BIVA	Bioimpedance vector analysis
BMI	Body mass index
BNP	Brain natriuretic peptide
BP	Blood pressure
CHF	Chronic heart failure
CI	Confidence interval
CKD	Chronic kidney disease
CVD	Cardiovascular disease
DM	Diabetes mellitus
ECW	Extracellular water
eGFR	Estimated glomerular filtration rate
ESKD	End-stage kidney disease
GN	Glomerulonephritis
HFpEF	Heart failure with preserved ejection fraction
HFrEF	Heart failure with reduced ejection fraction
HR	Hazard ratio
HTN	Hypertension
ICW	Intracellular water
IQR	Interquartile range
KDIGO	Kidney Disease Improving Global Outcomes
KRT	Kidney replacement therapy (haemodialysis, peritoneal dialysis or kidney transplant)
LL	Lower limit
LVEF	Left ventricular ejection fraction
MACE	Major adverse cardiac event
MRA	Mineralocorticoid receptor antagonist
MV	Multivariable
MVSA	Multivariable survival analysis
NOS	Not otherwise specified
NT-proBNP	N-terminal brain natriuretic peptide
NYHA	New York Heart Association class
OR	Odds ratio
PKD	Polycystic kidney disease
RASi	Renin-angiotensin system inhibitor
RJL	RJL Systems (manufacturer name)
sCr	Serum creatinine
T2DM	Type 2 diabetes mellitus
TBW	Total body water
uACR	Urinary albumin:creatinine ratio
UL	Upper limit
uPCR	Urinary protein:creatinine ratio
vasc	Vascular

Supplementary Table S1: PI(E)COS framework

Criteria	Definition
Population	Adult populations with heart failure and/or CKD
(Intervention/)Exposure	Fluid overload measured by whole-body bioimpedance analysis or spectroscopy
Comparison	Largely not applicable for observational studies. Studies may include, as a comparator, standard clinical assessment of fluid status used in routine care
Outcome(s)	All-cause mortality<br>Cardiovascular event or composite outcomes using study-specific definitions<br>Kidney disease progression using study-specific definitions
Study design	Observational and interventional studies

Supplementary Table S2: Summary of bioimpedance indices of fluid overload employed in included studies

	Units	Method	Represents	Reference ranges
**Absolute fluid overload**	Litres	BIS using Fresenius Body Composition Monitor (requires 3-compartment model)	Absolute measure of excess fluid volume, independent of body composition.	Manufacturer normal range: -1.1L to +1.1L. Authors have suggested additional threshold representing severe fluid overload >+2.5L.
**Relative fluid overload**	%	BIS using Fresenius Body Composition Monitor (requires 3-compartment model)	Excess fluid volume indexed to measured ECW volume allowing for comparison between individuals.	Not provided by manufacturer. Suggested by multiple authors: normal -7% to +7%, 7-15% mild and >15% severe fluid overload. Approximately equal to above absolute thresholds.
**Reactance Xc/H**	Ohms/metre (Ω/M)	Theoretically can be derived from all BIA & BIS devices	Raw impedance parameter measuring obstruction to the flow of electrical current where resistance relates to extra- and intracellular resistance & reactance relates to the cell membrane. Indexed to height in metres.	Not applicable.
**Resistance R/H**	Ohms/metre (Ω/M)	Theoretically can be derived from all BIA & BIS devices		
**Phase angle**	Degrees (°)	Theoretically can be derived from all BIA & BIS devices	Derived from a vector plot of reactance against resistance which describes the relationship between water resistance & cell resistance. Phase angle indicates the direction of the vector.	No existing reference ranges. Lower phase angle reflects higher degrees of fluid overload. Phase angle increases with increasing numbers of intact cells in the body and therefore also reflects other factors such as nutrition.
**BIVA hydration index**	%	Theoretically can be derived from all BIA & BIS devices	Derived from a vector plot of reactance against resistance presented as nomogram. Standard deviation ellipses are used to define normal ranges.	Dehydration <72.7%; normohydration 72.7-74.3%; fluid overload >74.3%. (1)
**Extracellular water (ECW)**	Litres	Multifrequency BIA & BIS devices	Volume of the extracellular water compartment.	Dependent upon age and sex. Approximately one third of total body water volume.
**ECW:total body water ratio** (or ECW:intracellular water ratio)	NA (ratio)	Multifrequency BIA & BIS devices	Ratio of ECW volume to other body fluid compartments: either as a fraction of the total body water volume or a ratio compared to the intracellular compartment.	0.36-0.39 considered normal by InBody device manufacturers. (2)

Supplementary Table S3: Associations between fluid overload and risk of kidney disease progression

Author	N	Follow-up (yrs)	Fluid overload definition	Baseline fluid overload Mean (SD)/ median (IQR)	Outcome definition	n outcomes	% outcomes	Outcomes/100 person yrs[1]	Outcome summary			Analysis	Covariates								HR/ OR	95% CI LL	95% CI UL
									Composite	KRT/ESKD	Δ eGFR		Age	Sex	DM	CVD	eGFR[2]	uACR	BP	Other			
**Bansal**	3751	7.0	Phase angle (◦) Quartile 1 vs quartiles 3 & 4 combined (<5.59 vs ≥6.4)	6.6 (1.8) ◦	>30% eGFR decline or ESKD (KRT)	1597	43	6.1	X	-	-	Cox MVSA	**X**	**X**						**Ethnicity, clinical site**	**1.78**	**1.56**	**2.04**
												Cox MVSA	X	X	X	X	X	X	X	Serum albumin; ethnicity, clinical site, smoking	0.99	0.86	1.14
**Liu**	1065	8.6	Absolute fluid overload (L) Tertiles: tertile 3 vs tertile 1[3]	1.3 (0.6-1.9) L	Worsening KDIGO CKD category (eGFR) and ≥25% eGFR decline	465	44	5.1	X	-	-	Cox MVSA	**X**	**X**	**-**	**-**	**-**	**-**	**-**	**Ethnicity**	**1.94**	**1.54**	**2.46**
												Cox MVSA	X	X	X		X	X	X	HbA1c, BMI, medication (RASi)	1.45	1.14	1.85
			Relative fluid overload (%); >7% vs ≤7%	10.2 (4.8-14.4) %	Worsening KDIGO CKD category (eGFR) and ≥25% eGFR decline	465	44	5.1	X	-	-	Cox MVSA	**X**	**X**	**-**	**-**	**-**	**-**	**-**	**Ethnicity**	**1.59**	**1.30**	**1.95**
												Cox MVSA	X	X	X		X	X	X	HbA1c, BMI, medication (RASi)	1.29	1.05	1.59
**Hung**	338	2.1	Absolute fluid overload (L) Per 1L increment	NA	(1) ≥50% eGFR decline or ESKD requiring chronic dialysis	100	30	14.1	X	-	-	Cox MVSA	**X**	**X**							**1.34**	**1.23**	**1.45**
												Cox MVSA	X	X	X	X			X	Medication (ACEi, ARB)	1.26	1.14	1.39
												Cox MVSA	X	X	X	X	X	X	X	Medication (ACEi, ARB)	1.25	1.11	1.41
			Relative fluid overload (%); ≥7% vs <7%	8.3 (8.6) %	(1) ≥50% eGFR decline or ESKD requiring chronic dialysis	100	30	14.1	X	-	-	Cox MVSA	**X**	**X**							**4.56**	**2.83**	**7.36**
												Cox MVSA	X	X	X	X			X	Medication (ACEi, ARB)	3.63	2.20	5.99
												Cox MVSA	X	X	X	X	X	X	X	Medication (ACEi, ARB)	2.44	1.44	4.13
					(2) eGFR slope analysis	NA	NA	NA	-	-	X	NA	Significantly greater eGFR decline in ≥7% vs <7%[4]										
**Khan**	312	1.0	ECW (L); assume per increment (not reported)	16.7 (3.7) L	KRT initiation[5]	36	12	11.5	-	X	-	MV logistic regression	X	X	X	-	X	X	X	Medication (ACEi/ARB/ diuretic)	3.25[6]	1.42	1.18[6]
**Tsai**	236	3.3	Relative fluid overload (%); >7% vs ≤7%	7.8 (8.6) %	(1) Dialysis initiation[7]	129	55	16.6	-	X	-	Cox MVSA	X	X	X	X	X	X	-	Medication (ACEi, ARB, diuretic, statin), LDL	1.53	1.02	2.28
					(2) Rapid eGFR decline >3ml/min/1.73m^2/year	88	37	11.3	-	-	X	MV logistic regression	X	X	X	X	X	X	-	Medication (ACEi, ARB, diuretic, statin), LDL	2.89	1.51	4.45
					(3) eGFR slope analysis	NA	NA	NA	-	-	X	NA. Significantly greater eGFR decline in >7% vs ≤7%[8]											
**Kohatsu**	194	1.4	ECW:TBW > median (0.48)	0.48 (0.04)	≥30% eGFR decline or ESKD (KRT or death)	107	55	39.4	X	-	-	Cox MVSA (figure only, HR not reported). "No significant difference" high vs low ECW:TBW groups											
**Schork**	179[10]	5.9	Absolute fluid overload (L) Per 1L increment	0.2 (-0.5 to 1.2) L	Progression to ESKD with KRT initiation[11]	33	19	3.2	-	X	-	Cox MVSA	-	-	X	-	X	X	X	NTpro-BNP	1.24	0.83	1.90
			Absolute fluid overload (L) >1L vs ≤1L		Progression to ESKD with KRT initiation[11]	33	19	3.2	-	X	-	Cox MVSA	-	-	X	-	X	X	X	NTpro-BNP	3.32	1.26	8.76
**Ohashi**	149	4.9	ECW:ICW; assumed per increment (not specified)	NA	≥50% eGFR decline or KRT initiation	52	35	7.1	X	-	-	Cox MVSA	X	-	X	-	X	X	X		1.15	1.03	1.26
**Esmeray**	100	1.0	Absolute fluid overload (L) >0.5L vs ≤0.5L	NA	ESKD requiring chronic dialysis	14	14	14.0	-	X	-	MV logistic regression	Not reported								1.76	1.20	2.57

Lower phase angle indicates higher degrees of fluid overload. Where more than one multivariable model is presented with different levels of adjustment, the preferred model is highlighted in bold. eGFR decline = from baseline. [1] Event rate calculated for all studies from N, n and years follow-up. [2] eGFR or other kidney function measure. [3] Tertiles assumed to be absolute fluid overload - not explicitly stated. [4] eGFR slope ≥7% vs <7%: -4.3 [-12.6, 1.2] vs -1.7 [-7.8, 2.7] mL/min/1.73m^2/year; p<0.05. [5] eGFR decline & death also reported but not by fluid overload. [6] Table reports HR but assumed to be OR - logistic regression analysis method; assume error in the UL of the 95% CI reported. [7] Transplant not mentioned however duplicate report of same study cites no transplant events. [8] eGFR slope presented overall & in 4 groups based upon fluid overload ≤7%/>7% & median NTpro-BNP: entire cohort -2.3(-4.1,-1.1); fluid overload ≤7% NT-proBNP ≤ median -1.8(-3.2,-0.9); fluid overload ≤7% NT-proBNP > median -1.6(-2.9,-0.9); fluid overload >7% NT-proBNP ≤ median -2.6(-5.4,-1.0); fluid overload >7% NT-proBNP >median -3.1(-6.1,-1.4) mL/min/1.73m^2/year; p<0.001. [10] 179 included, 177 with outcome data for kidney outcomes. [11] Also studied composite outcome progression to ESKD with KRT initiation or ≥30% eGFR decline however composite outcome not modelled with fluid overload.

Supplementary Table S4a: Study characteristics for all included heart failure cohorts

Author	Year	Region	Study design	N	Follow-up (years)	Heart failure inclusion criteria	CKD exclusion criteria/reporting	Method	Device	Outcomes reported		
										All-cause mortality	CV	Kidney
**Massari**	2019	Europe	Retrospective cohort	706	0.02	AHF; LVEF <40%, 40-49%, ≥50%	Included CKD	BIVA	CardioEFG (Akern)	N	Y	N
**Massari**	2020	Europe	Retrospective cohort	436	1.3	AHF & CHF; LVEF <40%, 40-49%, ≥50%	Included CKD	BIVA	CardioEFG (Akern)	Y	N	N
**Colin-Ramirez**	2012	North America	Retrospective cohort	389	3	CHF; HFrEF & HFpEF	Included CKD	BIA	Quantum X (RJL)	Y	N	N
**Di Somma**	2014	Europe	Prospective cohort	381[1]	0.1	AHF	Excluded eGFR <30	BIVA	Tetrapolar 50 kHz (Akern Srl)	N	Y	N
**Nunez**	2016	Europe	Prospective cohort	369	1	AHF	Included CKD	BIVA	CardioEFG (Akern)	Y	Y	N
**Lyons**	2017	North America	Prospective cohort	359	2.1	CHF; HFrEF & HFpEF	Not reported	BIA	InBody 520	N	Y	N
**Santarelli**, EHJ Acute CV Care	2017	Europe	Prospective cohort	336[2]	0.3	AHF	Included CKD	BIVA	EFG (Akern)	Y	Y	N
**Santarelli**, Intern Emerg Med	2017	Europe & South America	Prospective cohort	292	0.3	AHF	Included CKD	BIVA	EFG (Akern)	N	Y	N
**De Berardinis**	2014	Europe & North America	Prospective cohort	194	1.5	AHF	Excluded KRT; reported baseline CKD 31%	BIVA	Tetrapolar 50 kHz (Akern Srl)	Y	Y	N
**Sakaguchi**	2015	Asia	Prospective cohort	190[3]	0.5	AHF	Excluded sCr >3 mg/dL	BIA	BioScan 920-2 (Maltron Intl)	N	Y	N
**Liu**	2012	Asia	Randomised controlled trial	159[4]	0.5	AHF; LVEF <40%	Excluded sCr >5 mg/dL / nephritic; reported baseline CKD 38%	BIA	InBody 720	N	Y	N
**Siriopol**	2021	Europe	Prospective cohort	151	1.7	CHF; LVEF <45%	Excluded ESKD	BIS	Fresenius BCM	Y	N	N
**Koell**	2017	Europe	Prospective cohort	150	2	CHF; HFpEF (LVEF>50%)	Included CKD	BIS	Fresenius BCM	N	Y	N
**Soloveva**	2019	Russia	Prospective cohort	149	0.8	AHF	Excluded ESKD; reported baseline CKD 23%	BIVA	ABC-01 (Medass)	N	Y	N
**Trejo-Velasco**	2016	Europe	Prospective cohort	105	0.9	AHF	Included CKD	BIVA	CardioEFG (Akern)	N	Y	N
**Sakaguchi**	2020	Asia	Prospective cohort	100[5]	0.5	AHF	Included CKD	BIA	Bioscan 920-2 (Maltron Intl)	N	Y	N
**Curbelo**	2019	Europe	Prospective cohort	99	1	CHF	Excluded dialysis	BIA	Bodygram (Akern)	N	Y	N
**Villacorta**	2021	South America	Prospective cohort	80	0.6	AHF	Excluded dialysis (or imminent)	BIVA	EFG (Akern)	N	Y	N
**Alves**	2016	South America	Prospective cohort	71	2	AHF; LVEF ≤45%	Excluded serum creatinine >2.5 mg/dL or dialysis	BIA	Biodynamics 450	Y	N	N
**Di Somma**	2010	Europe	Prospective cohort	51[6]	0.3	AHF	Excluded eGFR <60	BIVA	NA	N	Y	N

[1] 270/381 with AHF; 111 controls. [2] 221/336 with AHF. [3] 130 with AHF + 60 hospitalized controls; controls used to determine predicted values ECW only, analysis is of AHF patients (not compared to controls). [4] 53 in case management with BIA group; 53 in case management without BIA; 53 controls (routine care). [5] 100 with central venous catheter and therefore included in survival analysis reporting fluid overload. [6] 25 AHF + 26 controls.

Supplementary Table S4b: Study characteristics for all included CKD cohorts

Author	Year	Region	Study design	N	Follow-up (yrs)	CKD inclusion criteria	Heart failure exclusion/reporting	Method	Device	Outcomes reported		
										All-cause mortality	CV	Kidney
**Bansal**	2018	North America	Prospective cohort	3751	7	eGFR 20-70	Excluded	BIA	Quantum II (RJL)	Y	Y	Y
**Liu**	2021	Asia	Prospective cohort	1065	8.6	CKD 1-4 & non-CKD with T2DM	Not excluded/reported	BIA	InBody S20	N	N	Y
**Vega**	2018	Europe	Prospective cohort	356	4.2	CKD 4-5	Included; reported baseline heart failure 27%	BIS	Fresenius BCM	Y	Y	N*
**Hung**	2015	Asia	Prospective cohort	338	2.1	CKD 3-5	Included in CVD outcome, baseline reported as CVD	BIS	Fresenius BCM	N	Y	Y
**Khan**	2017	Asia	Prospective cohort	312	1	CKD 3-5	Excluded	BIS	Fresenius BCM	N	N	Y
**Tsai**	2018	Asia	Prospective cohort	236	3.3	CKD 4-5	Included in outcome, baseline reported as CVD	BIS	Fresenius BCM	Y	Y	Y
**Kohatsu**	2021	Asia	Retrospective cohort	194	1.4	CKD 3-5	Not excluded/reported	BIA	BioScan 920-II	N	N	Y
**Schork**	2020	Europe	Retrospective cohort	179†	5.9	CKD 1-5	Not excluded/reported	BIS	Fresenius BCM	N	N	Y
**Caravaca**	2011	Europe	Prospective cohort	175	1.3	eGFR <40	Excluded	BIS	Fresenius BCM	Y	N	N
**Ohashi**	2015	Asia	Retrospective cohort	149	4.9	NOS	Included in CVD outcome, baseline not reported	BIA	InBody S20	Y	Y	Y
**Esmeray**	2018	Europe	Non-randomised experimental	100	1	CKD 3-4	Excluded	BIS	Fresenius BCM	Y	N	Y

* Event numbers reported for progression to dialysis however no analysis reported. †179 included, 177 with outcome data for kidney outcomes.

Supplementary Table S5a: Baseline participant characteristics from heart failure cohorts

Author	Year	Age, years	Male %	eGFR, ml/min/ $1.73m^2$	Serum creatinine mg/dL	BMI, kg/m^2	Systolic BP, mm/Hg	LVEF, %, mean (SD)	NYHA class III-IV %	Diabetes %	Hypertension %	Smoking %	Diuretic %	RASi %	NTpro-BNP/ BNP pg/ml	Haemo-globin g/dL	Serum albumin g/dL	Serum sodium mmol/L or mEq/L
**Massari**	2019	78 (10)	52	CrCl 46 (23)	1.6 (1.1)	28 (5)	-	44 (14)	-	24	-	-	Loop 97%	ACEi 36%; ARB 12%	830 (479-1810)*	12.0 (2.0)	3.2 (0.5)	139 (4)
**Massari**	2020	75 (11)	52	-	1.2 (0.8)	28 (5)	130 (25)	Preserved = 48%<br>Mid-range = 10%<br>Reduced = 42%	56	24	-	-	Loop 69%; MRA 69%	ACEi 39%; ARB 21%	503 (197-1000)*	13.0 (2.0)	3.3 (0.6)	139 (4)
**Di Somma†**	2014	77 (11)	53	57 (29)	-	27 (6)	140 (29)	-	-	37	79	-	-	-	717 (786)	-	-	-
**Nunez**	2014	73 (11)	50	-	1.2 (0.5)	-	148 (35)	49 (16)	27	46	78	11	Loop 97%; MRA 36%	ACEi 35%; ARB 30%	4041 (5921)	12.1 (1.9)	-	138 (4)
**Lyons**	2017	56 (14)	72	-	1.3 (1.1)	28 (6)	115 (19)	36 (16)	34	24	-	-	MRA 52%	84	334 (500)	-	4.7 (4.4)	-
**Santarelli,** EHJ Acute CV Care	2017	79 (8)	41	55 (26)	-	-	140 (27)	-	-	-	-	-	-	-	859 (985)	-	-	138 (5)
**De Berardinis**	2014	76 (11)	56	55 (29)	1.6 (1.1)	29 (14)	150 (34)	-	-	45	78	-	Loop 65%; MRA 13%	ACEi 28%; ARB 14%	873 (1024)	11.9 (0.0)	-	137 (64)
**Sakaguchi**	2015	74 (11)	55	49 (24)	1.2 (0.6)	-	137 (33)	45 (19)	-	37	71	-	Loop 67%; MRA 31%	56	653 (586)	12.0 (2.5)	3.7 (0.4)	141 (4)
**Siriopol**	2021	67(12)	70	67 (25)	1.1 (0.9-1.4) *	29 (5)	125 (18)	33 (10)	48	35	62	40	-	-	800 (400-1500)*	13.4 (2.1)	-	137 (5)
**Soloveva**	2019	69 (12)	70	54 (44-69)*	1.2 (1.0-1.5)	-	141 (28)	40 (14)	95	41	93	-	-	-	4046 (1956-5456)*	-	-	-
**Trejo-Velasco**	2016	69 (13)	56	-	1.1 (0.4)	-	127 (20)	39 (16)	-	44	78	49	Loop 98%; MRA 40%	76	4629 (3768)	12.4 (1.8)	-	136 (4)
**Sakaguchi**	2020	71 (12)	64	-	1.3 (0.8)	-	137 (34)	43 (18)	-	38	70	-	-	63	759 (599)	-	-	-
**Curbelo**	2019	84 (7)	41	55 (22)	1.2 (0.5)	27 (6)	126 (20)	58 (15)	26	34	93	-	Loop 84%; MRA 53%; Thiazide 18%	ACEi 35%; ARB 32%	1637 (2289)	12.7 (1.6)	-	140 (3)
**Alves**	2016	61 (12)	63	-	1.3 (0.4)	-	-	26 (8)	100	-	-	-	-	-	921 (545-1932)*	12.4 (2.0)	3.7 (0.5)	139 (4)

Limited to studies reporting characteristics for the full cohort. Baseline characteristics for entire cohort not available for Colin-Ramirez, Di Somma 2010, Koell, Liu, Santarelli & Villacorta studies. None of the heart failure studies reported urine protein. Data are presented as mean (SD) unless denoted by * which indicates median (IQR). † Baseline characteristics are for 270 with AHF who were studied in MV regression analysis.

Supplementary Table S5b: Baseline participant characteristics from CKD cohorts

**Author**	**Year**	**Age,** years	**Male %**	**eGFR,** ml/min/1.73m^2	**Urine protein**	**BMI,** kg/m^2	**Systolic BP,** mm/Hg	**Primary renal diagnosis, %**	**Diabetes %**	**Hypertension %**	**Smoking %**	**Diuretic %**	**RASi %**	**NTpro-BNP/ BNP** pg/ml	**Haemoglobin** g/dL	**Serum albumin** g/dL	**Serum sodium** mmol/L or mEq/L
**Liu**	2021	59 (9)	51	92 (69-103)*	uACR 21.6 (7.3-80.0) mg/g*	28 (5)	139 (18)	-	100	-	-	-	63	-	-	-	-
**Vega**	2018	67 (13)	64	16 (6)	Urine protein 0.5 (0.2-1.5) g/24h*	28 (5)	-	GN 23%; DM 19%; Vasc 28%; Interstitial 13%; PKD 10%; Other 7%	36	87	-	-	-	840 (370-1810)*	-	4.1 (0.4)	-
**Hung**	2015	66 (14)	69	29 (15)	uPCR 0.9 (0.3-2.5) g/g*	26 (4)	138 (17)	-	45	46	21	33	59	242 (78-771)*	-	3.6 (0.4)	136 (4)
**Khan**	2017	65 (6)	57	21 (9)	Urine protein >1 52%	24 (5)	140 (21)	-	64	86	31	-	57	-	-	4.2 (0.4)	139 (3)
**Tsai**	2018	65 (12)	53	16 (8)	uPCR 1.9 (2.1) mg/mg	24 (4)	138 (19)	-	39	81	18	22	53	262 (125-742)*	10.5 (1.8)	4.1 (0.4)	-
**Schork**	2020	60 (48-71)*	55	47 (30-71)*	uACR 43 (5-198) mg/g*	28 (26-32)*	134 (125-149)*	GN 38%; DM/ HTN 33%; Interstitial 2%; PKD 5%; Other 23%	21	81	-	61	78	182 (68-613)*	-	-	-
**Kohatsu**	2021	71 (12)	76	24 (11)	Urine protein 0.9 (0.2-2.2) g/24h*	25 (5)	133 (16)	GN 12%; DM 32%; Vasc 28%; Other 28%	47	94	-	32	68	-	11.4 (1.8)	3.9 (0.5)	-
**Caravaca**	2011	66 (14)	56	16 (6)	uACR 1.8 (2.1) mg/g	30 (5)	159 (24)	-	35	11†	-	52	-	-	12.0 (1.4)	4.1 (0.3)	-

Limited to studies reporting characteristics for the full cohort. Baseline characteristics for entire cohort not available for Bansal, Ohashi & Esmeray studies. None of the CKD studies reported left ventricular ejection fraction or NYHA class. Data are presented as mean (SD) unless denoted by * which indicates median (IQR). † Reported uncontrolled hypertension only.

Supplementary Table S6: Studies with multiple reports identified

	Author	Year	Journal	Title	N	Recruitment dates	Follow-up (yrs)	Fluid overload parameter	Outcomes
A	Tsai	2013	PLoS One	Is Fluid Overload More Important than Diabetes in Renal Progression in Late Chronic Kidney Disease?	472	Jan-Dec 2011	1.4	Relative fluid overload, %	KRT initiation; Δ eGFR
A	Tsai	2014	AJKD	Association of Fluid Overload With Kidney Disease Progression in Advanced CKD: A Prospective Cohort Study	472	Jan-Dec 2011	1.4	Absolute fluid overload, L	KRT initiation; Δ eGFR
A	Tsai	2015	CJASN	Association of Fluid Overload with Cardiovascular Morbidity and All-Cause Mortality in Stages 4 and 5 CKD	478	Jan-Dec 2011	1.9	Relative fluid overload, %	All-cause mortality; MACE
A	Tsai	2017	PLoS One	The interaction between fluid status and angiopoietin-2 in adverse renal outcomes of chronic kidney disease	290	Jan-Dec 2011	3.2	Absolute fluid overload, L	KRT initiation; Δ eGFR
**A**	**Tsai**	**2018**	**PLoS One**	**The interaction between N-terminal pro-brain natriuretic peptide and fluid status in adverse clinical outcomes of late stages of chronic kidney disease**	**239**	**Jan-Dec 2011**	**3.3**	**Relative fluid overload, %**	**All-cause mortality; MACE; KRT initiation; Δ eGFR**
**B**	**Hung**	**2015**	**J Am Heart Assoc**	**Volume Overload and Adverse Outcomes in Chronic Kidney Disease: Clinical Observational and Animal Studies**	**338**	**Sep 2011-Dec 2012**	**2.1**	**Relative fluid overload, %**	**Composite Δ eGFR/KRT; composite CV morbidity/ mortality**
B	Hung	2015	J Am Heart Assoc	Association of Fluid Retention With Anemia and Clinical Outcomes Among Patients With Chronic Kidney Disease	326		2.2	Relative fluid overload, %	
C	Khan	2016	PLoS One	Chronic Kidney Disease, Fluid Overload and Diuretics: A Complicated Triangle	312	NA	1.0	Absolute fluid overload, L	Δ eGFR; KRT initiation [1]
**C**	**Khan**	**2017**	**Clin Exper Nephrol**	**Diuretics prescribing in chronic kidney disease patients: physician assessment versus bioimpedence spectroscopy**	**312**	**NA**	**1.0**	ECW, L (absolute fluid overload, L)	Δ eGFR; KRT initiation
D	Orea-Tejeda	2010	Cardiology	Prognostic value of cardiac troponin T elevation is independent of renal function and clinical findings in heart failure patients	152	2002-2011	3.5	BIVA hydration index plot	All-cause mortality
**D**	**Colin-Ramirez**	**2012**	**Nutrition**	**Bioelectrical impedance phase angle as a prognostic marker in chronic heart failure**	**389**	**2002-2011 [2]**	**3.0**	**Phase angle, °**	**All-cause mortality**
D	Castillo-Martinez	2016	Nutrición hospitalaria	Body composition changes assessed by bioelectrical impedance and their associations with functional class deterioration in stable heart failure patients	275	2002-2011	0.5	Resistance/height, Ω/m	Change in NYHA class
E	Vega	2017	CKJ	Low lean tissue mass is an independent risk factor for mortality in patients with stages 4 and 5 non-dialysis chronic kidney disease	356	NA	1.8	Absolute fluid overload, L	All-cause mortality (CV events, KRT initiation) [3]
**E**	**Vega**	**2018**	**CKJ**	**Any grade of relative overhydration is associated with long-term mortality in patients with Stages 4 and 5 non-dialysis chronic kidney disease**	**356**	**From Jan 2011**	**4.2**	**Relative fluid overload, %**	**All-cause mortality**
F	Low	2021	J Diabetes Complicat	Higher extracellular water to total body water ratio was associated with chronic kidney disease progression in type 2 diabetes	1079	March 2011-March 2014	8.6	ECW:TBW ratio	Δ eGFR & worsening KDIGO category composite
**F**	**Liu**	**2021**	**Diab Res & Clin Practice**	**Association of overhydration and serum pigment epithelium-derived factor with CKD progression in diabetic kidney disease: A prospective cohort study**	**1065**	**March 2011-March 2014**	**8.6**	**Absolute (L) & (%) fluid overload**	**Δ eGFR & worsening KDIGO category composite**
**G**	**Ohashi**	**2015**	**J Nutr Health Aging**	**The associations of malnutrition and aging with fluid volume imbalance between intra- and extracellular water in patients with chronic kidney disease**	**149**	**2005-2009**	**4.9**	**ECW:ICW**	**All-cause mortality; CV events; Composite Δ eGFR/KRT**
G	Tai	2014	BMC Nephrol	Association between ratio of measured extracellular volume to expected body fluid volume and renal outcomes in patients with chronic kidney disease: a retrospective single-center cohort study	149	2005-2009	4.9	ECW:TBW	Composite Δ eGFR/KRT

The selected report from which data were extracted is highlighted in bold. Approach to selection as follows: maximal outcome data, maximal follow-up time, largest population. Where different fluid overload parameters were used, this was considered alongside the aforementioned factors and parameters most synonymous with other studies were favoured. [1] Multivariable survival analysis not reported by fluid overload (diuretic status only), author confirmed by email therefore 2017 report favoured. [2] Author confirmed all studies represent the same cohort and recruitment dates 2002-2011 provided by email; 2012 study favoured based upon population size and fluid overload measurement more synonymous with other studies. [3] 2017 paper states no association between fluid overload and KRT or CV events however not quantified. Several other included studies share authors but report upon distinct cohorts.

Supplementary Table S7: Risk of bias (ROB) assessment for all included studies

Study		1. Study Participation	2. Study Attrition	3. Prognostic Factor Measurement	4. Outcome(s) Measurement	5. Study Confounding	6. Statistical Analysis & Reporting
**Alves**	2016	L	M	L	L	M	L
**Bansal**	2018	L	L	L	L	L	L
**Caravaca**	2011	L	L	L	L	M	M
**Colin-Ramirez**	2012	L	L	L	L	M	M
**Curbelo**	2018	L	L	L	L	M	M
**De Berardinis**	2014	L	L	L	M	M	M
**Di Somma**	2010	L	L	L	L	M	M
**Di Somma**	2014	L	L	L	L	M	L
**Esmeray**	2018	L	L	L	M	M	M
**Hung**	2015	L	L	L	L	L	L
**Khan**	2017	L	L	L	L	L	M
**Koell**	2017	L	L	L	L	M	L
**Kohatsu**	2021	L	L	L	L	L	L
**Liu**	2012	L	L	L	L	L	L
**Liu**	2021	L	L	L	L	L	L
**Lyons**	2017	M	L	L	L	L	L
**Massari**	2019	L	L	L	L	M	M
**Massari**	2020	L	L	L	L	M	M
**Nunez**	2014	L	L	L	L	M	M
**Ohashi**	2015	M	L	L	L	L	M
**Sakaguchi**	2015	L	L	L	L	M	M
**Sakaguchi**	2020	L	L	L	L	M	M
**Santarelli,** EHJ Acute CV Care	2017	L	L	L	L	M	M
**Santarelli,** Intern Emerg Med	2017	L	L	L	M	M	M
**Schork**	2020	M	L	L	L	M	M
**Siriopol**	2021	L	L	L	L	M	M
**Soloveva**	2019	L	L	L	L	M	M
**Trejo-Velasco**	2016	L	L	L	L	M	M
**Tsai**	2018	L	L	L	L	L	L
**Vega**	2018	L	L	L	L	M	M
**Villacorta**	2021	L	L	L	L	M	M

L = low risk of bias; M = moderate risk of bias; H = high risk of bias. **Study participation**: All considered low ROB unless inclusion/exclusion criteria not adequately described. **Study attrition**: Retrospective studies are not subject to loss to follow-up therefore rated low ROB. Loss to follow-up rarely reported in included studies therefore assumed minimal if not reported and rated low ROB. Alves *et al.* table 2 reports 29 deaths & 30 survivors in overall cohort of N=71, outcome not reported for remaining 12. **Prognostic factor (fluid overload) measurement**: ROB depends on measurement being different for different levels of the outcome – all studies used the same measurement for all participants therefore rated low ROB. Some studies used fluid overload parameters which are subject to bias (ECW:ICW, ECW:TBW or inappropriate thresholds such as 0L absolute fluid overload) however because the same measurement was used for all participants, this does not introduce significant bias within the study. **Outcome measurement**: ROB assessment based upon whether outcome measurement is different related to baseline level of fluid overload therefore despite suboptimal methods in some studies, if these were not deemed likely to differ according to baseline fluid overload, studies were rated low ROB. **Study confounding & Statistical analysis & reporting**: confounding and statistical analysis commonly overlap and were therefore considered together. Observational studies are very likely to be subject to unmeasured confounding and studies generally adjusted for some, but not all, relevant factors therefore commonly rated moderate ROB. Where issues were identified with confounding variables/covariates, this is reflected in ROB assessment for domains 5 & 6.

Quality in Prognostic Studies (QUIPS) tool for risk of bias

1. Study Participation: The study sample adequately represents the population of interest

Consider the following:
a) The source population or population of interest is adequately described for key characteristics (CKD/HF history/eGFR, proteinuria, diabetes, CVD).
b) The sampling frame and recruitment are adequately described, including methods to identify the sample sufficient to limit potential bias (number and type used, e.g., referral patterns in health care)
c) Period of recruitment is adequately described
d) Place of recruitment (setting and geographic location) are adequately described
e) "Inclusion and exclusion criteria are adequately described (e.g., including explicit diagnostic criteria or “zero time” description)."
f) There is adequate participation in the study by eligible individuals
g) The baseline study sample (i.e., individuals entering the study) is adequately described for key characteristics (CKD/HF history/eGFR, proteinuria, diabetes, CVD).

High risk of bias: The relationship between fluid overload and outcome is very likely to be different for participants and eligible nonparticipants
Moderate risk of bias: The relationship between fluid overload and outcome may be different for participants and eligible nonparticipants
Low risk of bias: The relationship between fluid overload and outcome is unlikely to be different for participants and eligible nonparticipants

2. Study Attrition: The study data available (i.e., participants not lost to follow-up) adequately represent the study sample

Consider the following:
a) Response rate (i.e., proportion of study sample completing the study and providing outcome data) is adequate.
b) Attempts to collect information on participants who dropped out of the study are described.
c) Reasons for loss to follow-up are provided.
d) Participants lost to follow-up are adequately described for key characteristics (CKD/HF history/eGFR, proteinuria, diabetes, CVD).
e) There are no important differences between key characteristics (CKD/HF history/eGFR, proteinuria, diabetes, CVD) and outcomes in participants who completed the study and those who did not.

High risk of bias: The relationship between fluid overload and outcome is very likely to be different for completing and noncompleting participants
Moderate risk of bias: The relationship between fluid overload and outcome may be different for completing and noncompleting participants
Low risk of bias: The relationship between fluid overload and outcome is unlikely to be different for completing and noncompleting participants

3. Prognostic Factor (fluid overload) Measurement: The prognostic factor (fluid overload) is measured in a similar way for all participants

Consider the following:
a) A clear definition or description of fluid overload measurement is provided (method, device, parameter used, timing of measurement).
b) Method of fluid overload measurement is adequately valid and reliable to limit misclassification bias
c) Continuous variables are reported or appropriate cut-points (i.e., not data-dependent) are used.
d) The method and setting of measurement of fluid overload is the same for all study participants.
e) Adequate proportion of the study sample has complete data for fluid overload variable.
f) Appropriate methods of imputation are used for missing fluid overload data.

High risk of bias: The measurement of fluid overload is very likely to be different for different levels of the outcome
Moderate risk of bias: The measurement of fluid overload may be different for different levels of the outcome
Low risk of bias: The measurement of fluid overload is unlikely to be different for different levels of the outcome

4. Outcome(s) Measurement: The outcome(s) of interest is measured in a similar way for all participants

Consider the following:
a) A clear definition of outcome is provided, including duration of follow-up and level and extent of the outcome construct.

b) The method of outcome measurement used is adequately valid and reliable to limit misclassification bias
c) The method and setting of outcome measurement is the same for all study participants.

High risk of bias: The measurement of the outcome is very likely to be different by the baseline level of fluid overload
Moderate risk of bias: The measurement of the outcome may be different by the baseline level of fluid overload
Low risk of bias: The measurement of the outcome is unlikely to be different by the baseline level of fluid overload

5. Study Confounding: Important potential confounding factors are appropriately accounted for

Consider the following:
a) All important confounders (eGFR/CKD stage, HF type/EF, diabetes, proteinuria, BP etc), are measured.
b) Clear definitions of the important confounders measured are provided
c) Measurement of all important confounders is adequately valid and reliable
d) The method and setting of confounding measurement are the same for all study participants.
e) Appropriate methods are used if imputation is used for missing confounder data.
f) Important potential confounders are accounted for in the study design (e.g., matching for key variables, stratification, or initial assembly of comparable groups).
g) Important potential confounders are accounted for in the analysis (i.e., appropriate adjustment).

High risk of bias: The observed effect of fluid overload on the outcome is very likely to be distorted by another factor related to fluid overload and outcome
Moderate risk of bias: The observed effect of fluid overload on outcome may be distorted by another factor related to fluid overload and outcome
Low risk of bias: The observed effect of fluid overload on outcome is unlikely to be distorted by another factor related to fluid overload and outcome

6. Statistical Analysis and Reporting: The statistical analysis is appropriate, and all primary outcomes are reported

a) There is sufficient presentation of data to assess the adequacy of the analysis.
b) The strategy for model building (i.e., inclusion of variables in the statistical model) is appropriate and is based on a conceptual framework or model.
c) The selected statistical model is adequate for the design of the study.
d) There is no selective reporting of results.

High risk of bias: The reported results are very likely to be spurious or biased related to analysis or reporting
Moderate risk of bias: The reported results may be spurious or biased related to analysis or reporting
Low risk of bias: The reported results are unlikely to be spurious or biased related to analysis or reporting

MEDLINE (Ovid) search strategy

1. overhydration.ab,ti.
2. hyperhydration.ab,ti.
3. hypervol?emia.ab,ti.
4. fluid overload.ab,ti.
5. fluid status.ab,ti.
6. volume overload.ab,ti.
7. volume status.ab,ti.
8. (dry adj2 weight).ab,ti.
9. hydration.ab,ti.
10. congestion.ab,ti.
11. body composition.ab,ti.
12. bioimpedance.ab,ti.
13. bio-impedance.ab,ti.
14. bioelectrical impedance.ab,ti.
15. bio-electrical impedance.ab,ti.
16. extracellular.ab,ti.
17. phase angle.ab,ti.
18. exp electric impedance/
19. 1 or 2 or 3 or 4 or 5 or 6 or 7 or 8 or 9 or 10
20. 11 or 12 or 13 or 14 or 15 or 16 or 17 or 18
21. 19 and 20
22. (cardiovascular adj2 outcome$).ab,ti.
23. (cardiovascular adj2 event$).ab,ti.
24. (cardiovascular adj2 endpoint$).ab,ti.
25. (cardiovascular adj2 disease$).ab,ti.
26. (cardiac adj2 outcome$).ab,ti.
27. (cardiac adj2 event$).ab,ti.
28. (cardiac adj2 endpoint$).ab,ti.
29. (cardiac adj2 disease$).ab,ti.
30. (coronary adj2 disease$).ab,ti.
31. isch?emic heart disease$.ab,ti.
32. myocardial infarction.ab,ti.
33. myocardial isch?emia.ab,ti.
34. acute coronary syndrome.ab,ti.
35. heart failure.ab,ti.
36. cardiac failure.ab,ti.
37. stroke.ab,ti.
38. cerebrovascular accident.ab,ti.
39. cerebrovascular disease.ab,ti.
40. survival.ab,ti.
41. mortality.ab,ti.
42. death.ab,ti.
43. exp cardiovascular diseases/
44. exp heart diseases/
45. exp coronary disease/
46. exp myocardial ischemia/
47. exp heart failure/
48. exp stroke/
49. exp survival/
50. exp mortality/
51. exp death/
52. 22 or 23 or 24 or 25 or 26 or 27 or 28 or 29 or 30 or 31 or 32 or 33 or 34 or 35 or 36 or 37 or 38 or 39 or 40 or 41 or 42 or 43 or 44 or 45 or 46 or 47 or 48 or 49 or 50 or 51
53. ((egfr or gfr) adj slope).ab,ti.
54. ((egfr or gfr) adj3 decline).ab,ti.
55. ((egfr or gfr) adj3 change).ab,ti.
56. glomerular filtration rate slope.ab,ti.
57. (glomerular filtration rate adj3 decline).ab,ti.
58. (glomerular filtration rate adj3 change).ab,ti.
59. (kidney disease adj2 progression).ab,ti.
60. end stage kidney disease.ab,ti.
61. (kidney adj3 failure).ab,ti.

62. (kidney adj3 outcome$).ab,ti.
63. (kidney adj3 event$).ab,ti.
64. (kidney adj3 endpoint$).ab,ti.
65. (renal disease adj2 progression).ab,ti.
66. end stage renal disease.ab,ti.
67. (renal adj3 failure).ab,ti.
68. (renal adj3 outcome$).ab,ti.
69. (renal adj3 event$).ab,ti.
70. (renal adj3 endpoint$).ab,ti.
71. renal insufficiency.ab,ti.
72. anuria.ab,ti.
73. residual kidney function.ab,ti.
74. residual renal function.ab,ti.
75. (doubl$ adj3 creatinine).ab,ti.
76. renal replacement.ab,ti.
77. kidney replacement.ab,ti.
78. dialysis.ab,ti.
79. h?emodialysis.ab,ti.
80. renal transplant$.ab,ti.
81. kidney transplant$.ab,ti.
82. exp glomerular filtration rate/
83. exp renal insufficiency, chronic/
84. exp creatinine/
85. exp renal dialysis/
86. exp kidney transplantation/
87. 53 or 54 or 55 or 56 or 57 or 58 or 59 or 60 or 61 or 62 or 63 or 64 or 65 or 66 or 67 or 68 or 69 or 70 or 71 or 72 or 73 or 74 or 75 or 76 or 77 or 78 or 79 or 80 or 81 or 82 or 83 or 84 or 85 or 86
88. 52 or 87
89. 21 and 88

Kidney disease outcome nomenclature

A Kidney Disease: Improving Global Outcomes (KDIGO) Consensus Conference in 2019 reviewed the nomenclature used to describe kidney disease, aiming to achieve greater uniformity. The recommendations were published in 2020 (3)and it is expected the suggested nomenclature will, in time, replace the current commonly used terminology. This review preferentially used the newly proposed terms (for example, kidney failure, KF) in summarising findings however existing literature is likely to instead feature the terms end-stage kidney disease (ESKD) or end-stage renal disease (ESRD). The following table outlines how these terms are handled in this review.

**KDIGO 2020 nomenclature**	**Definition**	**Related terms**
Kidney failure (KF)	GFR <15 ml/min per 1.73 m^2 or treatment by dialysis For ≥3 months	End-stage kidney disease (ESKD)<br>End-stage renal disease (ESRD)<br>End-stage kidney failure (ESKF)<br>End-stage renal failure (ESRF)
Kidney replacement therapy (KRT)	Includes dialysis and transplantation	Renal replacement therapy (RRT)
Kidney failure with replacement therapy (KFRT)	CKD G5 treated by dialysis or CKD G1-G5 after transplantation; for epidemiologic studies, both should be included	ESKD/ESRD/ESKF/ESRF requiring dialysis/transplantation
Kidney failure without replacement therapy (CKD G5 without KRT)	CKD G5 where KRT is not chosen or not available	End-stage kidney disease (ESKD)<br>End-stage renal disease (ESRD)
Chronic kidney disease without KRT (CKD without KRT)	CKD G1–G5, A1–A3 of any cause, not receiving dialysis or transplantation	

References

1. Valle R, Aspromonte N, Milani L, Peacock FW, Maisel AS, Santini M, et al. Optimizing fluid management in patients with acute decompensated heart failure (ADHF): the emerging role of combined measurement of body hydration status and brain natriuretic peptide (BNP) levels. Heart Fail Rev. 2011;16(6):519-29.

2. Lyons KJ, Bischoff MK, Fonarow GC, Horwich TB. Noninvasive Bioelectrical Impedance for Predicting Clinical Outcomes in Outpatients With Heart Failure. Crit Pathw Cardiol. 2017;16(1):32-6.

3. Levey AS, Eckardt KU, Dorman NM, Christiansen SL, Hoorn EJ, Ingelfinger JR, et al. Nomenclature for kidney function and disease: report of a Kidney Disease: Improving Global Outcomes (KDIGO) Consensus Conference. Kidney Int. 2020;97(6):1117-29.
